# Comparative Analysis of Bacillus cereus Group Isolates' Resistance Using Disk Diffusion and Broth Microdilution and the Correlation between Antimicrobial Resistance Phenotypes and Genotypes

**DOI:** 10.1128/aem.02302-21

**Published:** 2022-03-22

**Authors:** Emma Mills, Erin Sullivan, Jasna Kovac

**Affiliations:** a Department of Food Science, The Pennsylvania State University, University Park, Pennsylvania, USA; Centers for Disease Control and Prevention

**Keywords:** *Bacillus cereus* group, antimicrobial resistance, antimicrobial resistance genes, disk diffusion, broth microdilution, sensitivity, specificity

## Abstract

Bacillus cereus group isolates (*n* = 85) were screened for phenotypic resistance to 18 antibiotics using broth microdilution and CLSI M45 *Bacillus* spp. breakpoints. The susceptibility to 9 out of 18 antibiotics was also tested using disk diffusion method and M100 Staphylococcus spp. breakpoints when available. Overall, a high prevalence of susceptibility to clinically relevant antibiotics was identified using broth microdilution. For most tested antibiotics, a poor correlation was found between zones of inhibition and MICs. Using the broth microdilution results as a reference for comparison, we identified high error rates and low categorical agreement between results produced using disk diffusion and broth microdilution for the seven tested antibiotics with defined breakpoints. This suggests that disk diffusion should be avoided for AST of B. cereus group isolates. Further, we detected antimicrobial resistance genes with ARIBA and ABRIcate to calculate the sensitivity and specificity for predicting phenotypic resistance determined using broth microdilution based on the presence of detected antimicrobial resistance genes (ARGs). ARGs with poor sensitivity and high specificity included *rph* (rifampicin, 0%, 93%), *mph* (erythromycin, 0%, 99%), *bla1* (penicillin, 29%, 100%), and *blaZ* (penicillin, 56%, 100%). Compared to penicillin, *bla1* and *blaZ* had lower specificity for the prediction of ampicillin resistance. Overall, none of the ARGs had both high sensitivity and specificity, suggesting the need for further study of the mechanisms underlying phenotypic antimicrobial resistance in the B. cereus group.

**IMPORTANCE** Bacillus cereus group includes human pathogens that can cause severe infections requiring antibiotic treatment. Screening of environmental and food isolates for antimicrobial resistance can provide insight into what antibiotics may be more effective therapeutic options based on the lower prevalence of resistance. Currently, the comparison of antimicrobial susceptibility testing results using the disk diffusion method is complicated by the fact that many previous studies have used Staphylococcus spp. breakpoints to interpret their results. In this study, we compared the results of disk diffusion interpreted using the Staphylococcus spp. breakpoints against the results of broth microdilution interpreted using *Bacillus* spp. breakpoints. We demonstrated that the disk diffusion method does not produce reliable results for B. cereus group isolates and should therefore be avoided. This study also provides new insight into poor associations between the presence of antimicrobial resistance genes and resistance phenotypes for the B. cereus group.

## INTRODUCTION

Bacillus cereus group, also known as Bacillus cereus sensu lato (*s.s.*), comprises spore-forming, Gram-positive rod-shaped bacteria commonly found in the environment and food (1–5). The B. cereus group is composed of eight genomospecies that are differentiated among each other based on the non-overlapping genome average nucleotide identity (ANI) of 95.2 (6). The B. cereus group genomospecies include B. pseudomycoides, *B. paramycoides*, *B. mosaicus*, B. cereus sensu stricto (*s.s.*), *B. toyonensis*, B. mycoides, *B. cytotoxicus*, *B. luti* (6). These eight genomospecies are phylogenetically separated into eight phylogenetic groups, defined based on the *panC* gene polymorphisms, that are consistent with the genomospecies taxonomy (7).

The B. cereus group species vary in their cytotoxicity toward human cells, which can be used as an indicator of pathogenicity. Bacillus cereus species (Bacillus cereus *s.s.*), which is known as a foodborne pathogen, has been reported to be overrepresented by strains with cytotoxic capability (8, 9).

Scallan et al. (2011) estimated that 63,400 cases of Bacillus cereus foodborne illness occur in the United States annually (3). The prevalence of infections with B. cereus group bacteria may be underestimated due to the nature of foodborne illnesses and the passive surveillance of B. cereus. Although foodborne illness cases caused by B. cereus tend to be self-limiting, severe infections reported in the past required hospitalization and were lethal (10–13). For example, a 2003 outbreak linked to pasta salad contaminated with B. cereus consisting of 5 children led to one fatality (10). In 2008, another fatal case of B. cereus infection was reported due to the consumption of contaminated pasta (11). B. cereus was also reported to cause other types of severe infections. In a retrospective study conducted at a French university hospital between 2008 and 2012, they found that nearly 30% of 57 patients hospitalized due to B. cereus infection had bacteremia and 28% had skin infection (14). Of the 57 hospitalized patients, 12% had died (14). Others have also reported B. cereus group infections associated with the hospital environment and supplies, including catheter-associated bloodstream infections in children in Japan and contaminated health care kits associated with cutaneous anthrax-like infections in newborns in India (15, 16). Although most B. cereus group infections may not require antimicrobial treatment, it is important to prepare for those that warrant critical care, particularly if novel infectious strains emerge within the B. cereus group.

The Centers for Disease Control and Prevention recommend ciprofloxacin, doxycycline, and β-lactam antibiotics for treatment of inhalation exposure to B. anthracis (genomospecies *B. mosaicus* biovar Anthracis = B. Anthracis) (17). Severe non-anthrax Bacillus cereus infections are commonly treated with vancomycin, gentamicin, linezolid, levofloxacin, and clindamycin antibiotics (18–21). These antibiotics are widely used clinically due to the high prevalence of β-lactamases found among Bacillus cereus group isolates (22–24), although specific β-lactams (e.g., carbapenems) are also used in combination for the treatment of clinical cases (18, 25). Notably, there are reports of poor patient outcomes due to carbapenem resistance (12, 26). Recent studies have shown a high prevalence of resistance against penicillin, early cephalosporins, and 3rd and 4th generation cephalosporins among environmental isolates (2, 27–29). There have also been reports of a high prevalence of trimethoprim-sulfamethoxazole resistance in environmental isolates from Germany, China, and Ghana (2, 27, 29). Understanding the prevalence of antimicrobial resistance among B. cereus group isolates can inform the selection of antibiotics for therapeutic use and potentially improve treatment outcomes.

In addition to understanding the prevalence of antimicrobial nonsusceptibility phenotypes, the information about underlying mechanisms of phenotypic antimicrobial resistance is critical for the development of rapid diagnostic assays and for the surveillance of antimicrobial resistance. Studies on other foodborne pathogens, such as Salmonella and Campylobacter, have demonstrated the existence of a strong relationship between antimicrobial resistance genes (ARG) or resistance mutations in housekeeping genes and phenotypic antibiotic resistance (30, 31). However, this relationship is yet to be characterized for the broad Bacillus cereus group, beyond B. anthracis (17).

To study the correlation between individual ARGs and phenotypic resistance, isolates’ susceptibility needs to be determined using standard methods and resistance interpretation breakpoints. Two commonly used methods for phenotypic antimicrobial susceptibility testing include disk diffusion and broth microdilution, which provide information on the zones of inhibition or MICs, respectively. The interpretation of the results of disk diffusion for B. cereus isolates is challenged by the fact that current CLSI M45 guidelines only provide breakpoints for broth microdilution for *Bacillus* spp. (excluding B. anthracis). When utilizing the disk diffusion method, many have therefore applied Staphylococcus spp. CLSI M100 breakpoints for the interpretation of zones of inhibition for B. cereus group isolates (27, 29, 32). It is unclear whether the disk diffusion results interpreted using the S. aureus resistance breakpoints (CLSI M100) produce results consistent with the results of broth microdilution (i.e., gold standard method) interpreted using *Bacillus* spp. breakpoints defined in the CLSI M45. Hence, we sought to evaluate the consistency of resistance interpretation for 85 B. cereus isolates using disk diffusion results interpreted using S. aureus breakpoints (CLSI M100) and broth microdilution results interpreted using *Bacillus* spp. breakpoints (CLSI M45). We further assessed the relationship between the detected ARGs and broth microdilution-based antimicrobial resistance phenotypes to determine whether individual antimicrobial resistance genes are sensitive and specific markers of phenotypic resistance.

## RESULTS AND DISCUSSION

### The tested isolate collection was phylogenetically diverse and represented by five B. cereus group species.

A total of 85 Bacillus cereus group isolates were available in our culture collection and were included in this study. These isolates had been previously collected from dairy-associated environments (65%), food processing environments (27%), and natural environments (7%) between 2002 to 2014, mostly from New York state (89%). All isolates and the corresponding metadata are listed in Table S1. Whole-genome sequences for 85 isolates were obtained from the NCBI SRA database and were assembled (8, 33, 34). The average N50 of assembled genomes was 217 Kbp, the average genome size was 5.6 Mbp, and the average number of contigs longer than 1,000 bp was 129 contigs (supplemental material, Table S1).

Isolates were taxonomically identified using the average nucleotide identity (ANI) method implemented in the BTyper3 (36). Isolates belonged to 5 different species and the most abundant species were *B. mosaicus* (33%), B. mycoides (27%), and B. cereus *s.s.* (24%) (Table S1). Consistent with the species identification, most isolates were classified in *panC* phylogenetic groups VI *mycoides/paramycoides* (31%), II *mosaicus/luti* (26%), and IV *cereus sensu stricto* (26%) (supplemental material, Table S1). There was a high MLST sequence type (ST) diversity among isolates, with 62 unique ST identified (supplemental material, Table S1). Among these, 80% of identified STs were singletons. The most abundant STs were 1099 (*n* = 6), 1272 (*n* = 4), 222 (*n* = 4) and 410 (*n* = 3), and 1346 (*n* = 3), which belonged to phylogenetic groups IV *cereus s.s.*, II *mosaicus/luti*, VI *mycoides/paramycoides*, and I *pseudomycoides*, respectively (supplemental material, Table S1). The 5 isolates carrying Bt toxin-encoding genes (i.e., defined as biovar Thuringiensis) belonged to three species, B. cereus *s.s.* biovar Thuringiensis (*n* = 2), B. mycoides biovar Thuringiensis (*n* = 2), and *B. mosaicus* biovar Thuringiensis (*n* = 1).

In addition to genetic diversity, we also examined the prevalence of genes encoding for three major enterotoxins that have previously been associated with toxicoinfection caused by B. cereus group isolates (35). These include the nonhemolytic enterotoxin encoded by an operon *nheABC*, the hemolysin BL encoded by an operon *hblABCD*, and the cytotoxin K encoded by *cytK*, all of which are proteinaceous pore-forming enterotoxins (35). All isolates carried at least one gene from the nonhemolytic enterotoxin *nheABC* operon, which is similar to reports from Ghana and China where they had also reported a high prevalence of *nhe* genes in isolates obtained from dairy-associated environments and ready-to-eat foods, respectively (2, 36) (supplemental material, Table S1). In 13 isolates’ genomes, we did not detect any *hbl* genes, whereas 65 isolates carried *hblABCD*, 6 carried *hblCD*, and only one carried *hblACD* (Table S1), alike abundance reported in previous studies (2, 36). A total of 40% of tested isolates harbored a gene encoding cytotoxin K-2, which is similar to the prevalence reported in Germany (37%) (29). It is important to note that the prevalence of virulence genes may be underreported in our study due to the use of draft genomes for gene detection.

### Poor correlation between zones of inhibition and MICs demonstrates that the disk diffusion method is unsuitable for Bacillus cereus group antimicrobial susceptibility testing.

**FIG 1:**
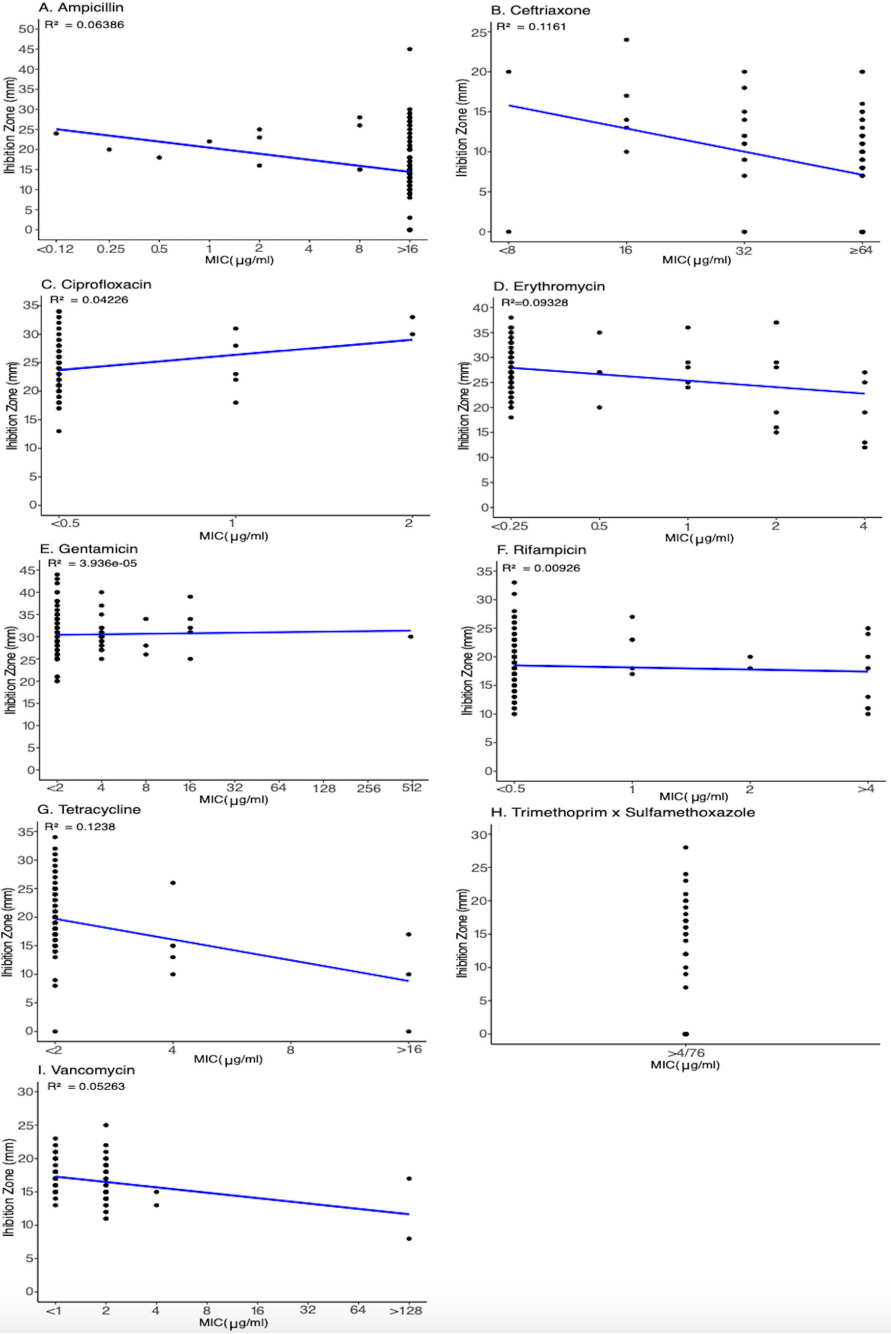
Scatterplots showing the analysis of zones of inhibition produced by disk diffusion method (Y axis) and MICs produced by broth microdilution method (X axis) for antibiotics (A) ampicillin, (B) ceftriaxone, (C) ciprofloxacin, (D) erythromycin, (E) gentamicin, (F) rifampicin, (G) tetracycline, (H) trimethoprim-sulfamethoxazole, and (I) vancomycin. R^2^ values for each antibiotic tested are shown below an antibiotic name.

Nine antibiotics (i.e., ampicillin, ceftriaxone, ciprofloxacin, erythromycin, gentamicin, rifampicin, tetracycline, trimethoprim-sulfamethoxazole, and vancomycin) were tested and compared using disk diffusion and broth microdilution assays to determine the relationship between these two methods (Fig. 1). We found an overall poor correlation between the results of the two methods, which was reflected in low R^2^ values for nine tested antibiotics. R^2^ values ranged from the lowest of 3.9 × 10^−5^ for gentamicin to the highest of 0.1238 for tetracycline. Since all MIC values for trimethoprim in combination with sulfamethoxazole were >4/76 (μg/mL), the R^2^ could not be calculated for this combination of antibiotics. Although the disk diffusion method has often been used for determining antimicrobial susceptibility of Bacillus cereus group isolates (27, 29, 36, 37), the CLSI M45 manual only recommends the broth dilution method for the most reliable susceptibility testing (38). Our findings are consistent with this guideline as we found a poor correlation between zones of inhibition obtained with disk diffusion and MICs obtained with broth microdilution. We, therefore, advise against the use of the disk diffusion method for susceptibility testing of B. cereus group isolates.

### High error rates suggest that the disk diffusion test with results interpreted using Staphylococcus spp. breakpoints should be avoided when testing the antimicrobial susceptibility of B. cereus group isolates.

Seven antibiotics (i.e., ampicillin, ciprofloxacin, erythromycin, gentamicin, rifampicin, tetracycline, trimethoprim-sulfamethoxazole) for which CLSI M100 Staphylococcus spp. and CLSI M45 *Bacillus* spp. breakpoints have been defined, were included in the analysis to determine the error rates and categorical agreement between disk diffusion and broth microdilution methods (Table 1). The results of broth microdilution interpreted with CLSI M45 *Bacillus* spp. breakpoints were used as a reference when comparing with the results of disk diffusion interpreted with M100 Staphylococcus spp. breakpoints. Minor, major, and very major error rates were calculated. A minor error is an error that occurs when one method reports an intermediate result, and the other method reports a susceptible or resistant outcome. A major error occurs when disk diffusion testing reports a resistant result, and the broth microdilution reports a susceptible result. A very major error occurs when the disk diffusion reports a susceptible result, and the broth microdilution reports a resistant result. The minor and major errors occurred most frequently when isolates were tested for susceptibility to tetracycline (35%) and rifampicin (34%), respectively. A very major error occurred most frequently when isolates were tested for susceptibility to ampicillin (35%). Consistent with these findings, the categorical agreement (i.e., % of isolates for which both methods produced the same result) was lowest for rifampicin (39%), tetracycline (54%), and ampicillin (65%). In contrast, the highest categorical agreement was obtained for gentamicin (88%), ciprofloxacin and trimethoprim-sulfamethoxazole (78%), and erythromycin (74%). Overall, these results indicate that the application of Staphylococcus spp. breakpoints, although commonly used for determining antimicrobial susceptibility of Bacillus cereus group isolates (27, 29, 36, 37), can cause inaccurate interpretation of antimicrobial susceptibility and should therefore be avoided. Instead, the broth microdilution method is advised to be used for antimicrobial susceptibility testing of B. cereus group isolates. The results of broth microdilution may be interpreted using the CLSI M45 guideline (38).

**TABLE 1:** Error rates and categorical agreement for antimicrobial susceptibility testing results of B. cereus group isolates obtained using disk diffusion and broth microdilution (reference) methods

	Percent (count)				
Antibiotic	Minor error	Major error	Very major error	Categorical agreement (%)	Total
Ampicillin	0	0	35 (30)	65 (55)	85
Ciprofloxacin	19 (16)	1 (1)	2 (2)	78 (66)	85
Erythromycin	26 (22)	0	0	74 (63)	85
Gentamicin	4 (3)	0	8 (7)	88 (75)	85
Rifampicin	24 (20)	34 (29)	4 (3)	39 (33)	85
Tetracycline	35 (30)	11 (9)	0	54 (46)	85
Trimethoprim-sulfamethoxazole	7 (6)	0	15 (13)	78 (66)	85

### The prevalence of resistance to clinically relevant antibiotics levofloxacin, clindamycin, and linezolid was low.

**FIG 2:**
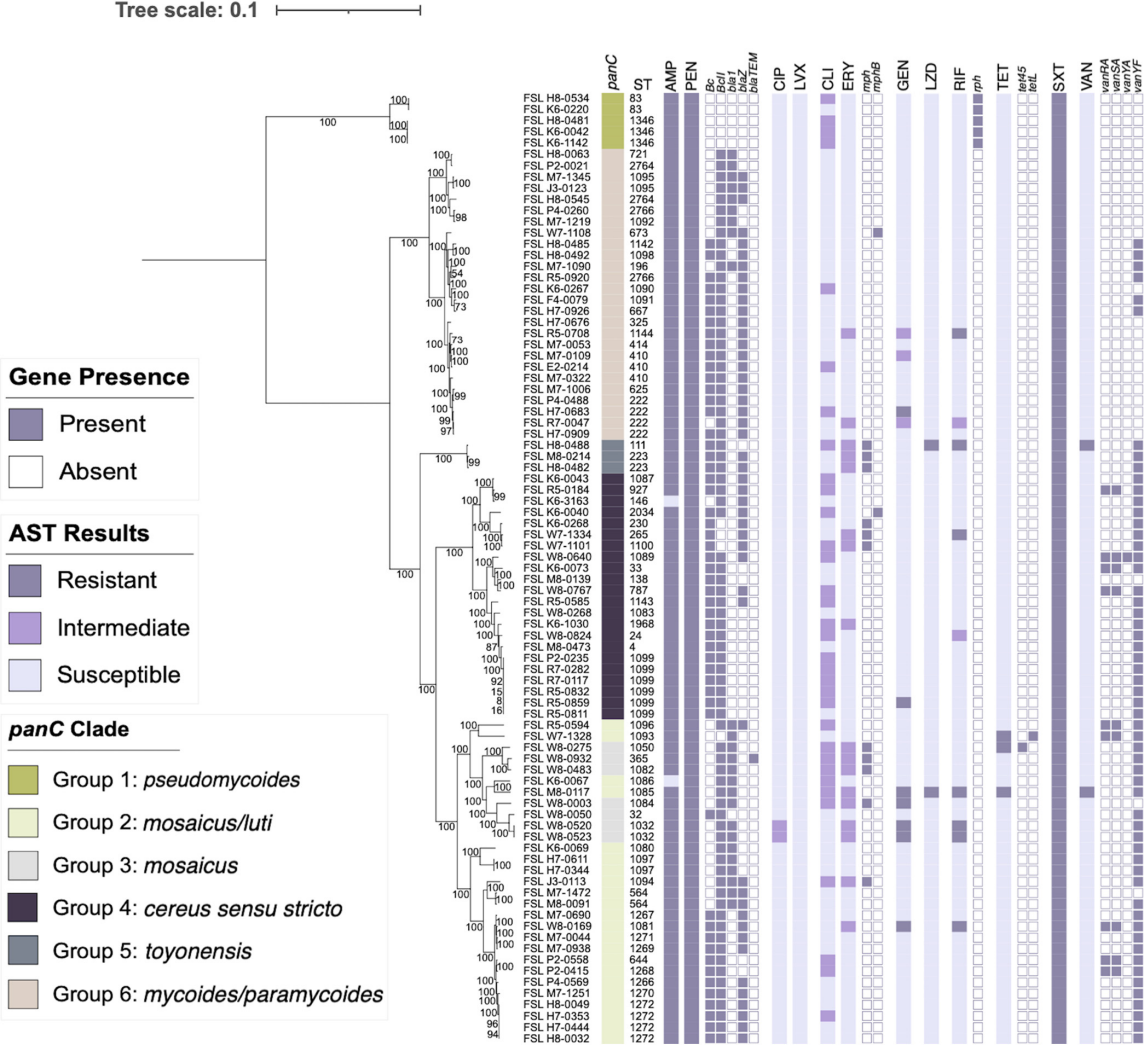
Phylogenetic tree for 85 B. cereus group isolates, annotated with the *panC* phylogenetic grouping (*panC*), MLST sequence type (ST), phenotypic resistance to ampicillin (AMP), penicillin (PEN), ciprofloxacin (CIP), levofloxacin (LVX), clindamycin (CLI), erythromycin (ERY), gentamicin (GEN), linezolid (LZD), tetracycline (TET), trimethoprim-sulfamethoxazole (SXT), and vancomycin (VAN). Phenotypic resistance was tested using the broth microdilution assay and interpreted using CLSI M45 *Bacillus* spp. breakpoints, except for linezolid for which EUCAST v12.0 *Bacillus* spp. breakpoints were used. The genes listed next to each antibiotic have previously been linked with phenotypic resistance to these antibiotics.

A total of 12 of the 18 antibiotics tested using broth microdilution had defined clinical breakpoints in either CLSI M45 (i.e., ampicillin, ciprofloxacin, clindamycin, erythromycin, gentamicin, levofloxacin, penicillin, rifampicin, tetracycline, trimethoprim-sulfamethoxazole, and vancomycin) or EUCAST v12.0 (i.e., linezolid) for *Bacillus* spp. All AST results are reported in the Supplemental Material, Table S2 and the distributions of MICs for these antibiotics are shown in the Supplemental Material, Fig. S1. Susceptibility testing revealed no or low prevalence of resistance to antibiotics clindamycin (0%), ciprofloxacin (0%), erythromycin (0%), gentamicin (8%), levofloxacin (0%), linezolid (2%), rifampicin (8%), tetracycline (4%), and vancomycin (2%) (Table 2). The seven gentamicin and seven rifampicin-resistant isolates were diverse and belonged to different phylogenetic groups and MLST sequence types (Fig. 2). Two of the three tetracycline-resistant isolates belonged to phylogenetic group II *mosaicus/luti* (Fig. 2). The two isolates resistant to linezolid and vancomycin belong to phylogenetic groups II *mosaicus/luti* and V *toyonensis.* Although no isolates were identified as resistant to clindamycin, 39% (*n* = 33) were found to be intermediate (Table 2). Nearly half (42%) of all clindamycin intermediate isolates belonged to phylogenetic group IV *cereus sensu stricto* (Fig. 2). Similarly, there were no erythromycin-resistant isolates, but 20% were identified as intermediate and were found among different phylogenetic groups (Fig. 2). All or nearly all isolates were resistant to ampicillin (98%), penicillin (100%), and trimethoprim-sulfamethoxazole (100%) (Table 2). While we describe to which phylogenetic groups resistant isolates belong, we did not carry out a statistical analysis of associations, because our data set does not meet the conditions for the validity of chi-square analysis. Specifically, the expected counts were less than 5 in some categories due to some phylogenetic groups being represented by fewer than 5 isolates (e.g., group 1, 5) and/or specific resistance genes or phenotypes being observed in fewer than 5 isolates per clade.

**TABLE 2:** Prevalence of antimicrobial resistance among 85 tested B. cereus group isolates

Antibiotic	Interpretation breakpoints	Percentage (count)		
		R^*a*^	I^*b*^	S^*c*^
Linezolid	EUCAST v12.0 *Bacillus* spp.	2 (2)	0	98 (83)
Ampicillin	M45 CLSI *Bacillus* spp.	98 (83)	0	2 (2)
Ciprofloxacin	M45 CLSI *Bacillus* spp.	0	2 (2)	98 (83)
Clindamycin	M45 CLSI *Bacillus* spp.	0	39 (33)	61 (52)
Erythromycin	M45 CLSI *Bacillus* spp.	0	20 (17)	80 (68)
Gentamicin	M45 CLSI *Bacillus* spp.	8 (7)	4 (3)	88 (75)
Levofloxacin	M45 CLSI *Bacillus* spp.	0	0	100 (85)
Penicillin	M45 CLSI *Bacillus* spp.	100 (85)	0	0
Rifampicin	M45 CLSI *Bacillus* spp.	8 (7)	2 (2)	89 (76)
Tetracycline	M45 CLSI *Bacillus* spp.	4 (3)	0	96 (82)
Trimethoprim/Sulfamethoxazole	M45 CLSI *Bacillus* spp.	100 (85)	0	0
Vancomycin	M45 CLSI *Bacillus* spp.	2 (2)	0 (0)	98 (83)

a R, resistant.

b I, intermediate.

c S, susceptible.

In terms of resistance prevalence, the prevalence of resistance to clinically relevant antibiotics, such as vancomycin, levofloxacin, clindamycin, and linezolid was low. This is consistent with the reports that the use of these antibiotics for the treatment of B. cereus group infections leads to positive clinical outcomes (19–21, 39). A group in Ghana used broth microdilution to test antimicrobial susceptibility of 96 dairy-associated Bacillus cereus group isolates, which allows for direct comparison of their report with the results from the present study (2). Similar to our results, Owusu-Kwarteng and colleagues identified all isolates susceptible to ciprofloxacin, gentamicin, rifampin, tetracycline, and vancomycin, and all isolates resistant to ampicillin and penicillin (2). They identified high trimethoprim-sulfamethoxazole resistance prevalence (80%), however the resistance prevalence in our study was greater (2). Lastly, higher prevalence of susceptibility to clindamycin (100%) was identified among isolates studied in Owusu-Kwarteng et al., compared to isolates studied here (61%) (2).

The 6 remaining antibiotics (i.e., ceftriaxone, daptomycin, gatifloxacin, oxacillin, quinupristin-dalfopristin, and streptomycin) tested with broth microdilution did not have defined clinical breakpoints for *Bacillus* spp. in CLSI or EUCAST guidelines. Definitions of ecological cutoffs were attempted using EUCAST ECOFFinder; however, due to our skewed distributions and the fact that our data set does not meet the criteria outlined by EUCAST, ecological cutoff values were left undetermined (40, 41). The distributions of MICs for these antibiotics are shown in Supplemental Material, Fig. S2.

### ABRIcate in conjunction with MEGARes 2.0 database resulted in the detection of the largest number of antimicrobial resistance genes.

Antimicrobial resistance genes were detected using both unassembled reads with ARIBA v2.14.6 and assembled genomes with ABRIcate v1.01., in conjunction with both MEGARes 2.0 and ResFinder 4.1 databases. ARGs detected in the 85 B. cereus group isolates are reported in Supplemental Material, Table S3. ABRIcate in conjunction with MEGARes 2.0 and ResFinder detected a median of 4 and 2 ARGs per isolate, respectively (supplemental material, Table S3). A search with ARIBA in conjunction with MEGARes 2.0 and ResFinder 4.1 databases resulted in a median of 2 and 0 detected ARGs per isolate, respectively. Given that the largest number of ARGs was detected using ABRIcate in conjunction with MEGARes 2.0 database, the genes identified by this program and database combination were used in further analysis (supplemental material, Table S3). The detection of acquired antimicrobial resistance genes by two different programs in conjunction with two different databases resulted in a different number of detected genes. Premkrishnan et al. also identified discrepancies in the number of detected antimicrobial resistance genes when using different methods for the detection of ARGs (42). This finding shows the importance of using multiple ARG detection tools to improve the detection of antimicrobial resistance genes (42).

### β-lactamase-encoding genes were detected in most isolates while other resistance genes such as *tet* and *mph* were less prevalent.

Nearly all isolates (93%) harbored intrinsic antimicrobial resistance genes *Bc* (*n* = 53) and/or a *BcII* (*n* = 76) that encode zinc metallo-β-lactamases. These genes are known to confer resistance to penicillins, carbapenems, and cephalosporins in B. cereus group species (Fig. 1) (23, 43, 44). Of the six isolates that did not have these genes detected, five belonged to *panC* phylogenetic group I (*pseudomycoides*) (Fig. 2). Within these five group I (*pseudomycoides*) isolates, the only gene identified was *rph* (rifampin phosphotransferase). The effect of this gene on rifampicin resistance in Bacillus cereus is still unclear. Three additional β-lactamase-encoding genes were identified among 85 tested isolates, including *bla1* (*n* = 25), *blaZ* (*n* = 48), and *blaTEM* (*n* = 1, ST-365) (Supplemental Material, Table S3). *bla1* was only identified in half of the *panC* clades including II *mosaicus/luti*, III *mosaicus*, VI *mycoides/paramycoides* (Fig. 2). *blaZ* was sporadically scattered among most clades and sequence types (Fig. 2). All isolates carried a *fosB*, a thiol transferase-encoding gene that was previously associated with fosfomycin resistance in other bacterial species (22) (Supplemental Material, Table S3); however, the phenotypic resistance to fosfomycin was not tested as this antibiotic was not included in the Sensititre plate antibiotic panel. Erythromycin resistance-conferring genes *mphB* (*n* = 2) and *mph* (*n* = 11) were mostly identified in groups III *mosaicus* (*n* = 4), IV *cereus sensu stricto* (*n* = 4), and VI *toyonensis* (*n* = 3) (Fig. 2) (29). Only two isolates carried tetracycline predicting genes, *tet45* or *tetL*, found in groups III *mosaicus* and II *mosaicus/luti* respectively (Fig. 2). Although van genes, *vanRA* (*n* = 9), *vanSA* (*n* = 9), *vanYA* (*n* = 1), and *vanYF* (*n* = 57), were identified in our collection, none were associated with vancomycin resistance (Fig. 2) (45). In order to confer vancomycin resistance an isolate must have all *vanM/vanB* glycopeptide resistance genes present (46).

Only a few published studies have focused on characterizing the presence and abundance of acquired ARGs in B. cereus group isolates (29, 47, 48). Bianco et al. investigated the prevalence of ARG in a collection of 17 B. cereus group clinical blood isolates (47). Similar to what we report for our isolate collection, they reported a high prevalence of β-lactamase-encoding genes, specifically *bla-1* and *bla-2* (48). In our isolates collection, we found a substantially higher prevalence of the *blaZ* compared to the prevalence of this gene among the 17 clinical isolates studied in Bianco et al. (47). In our study, the Bianco et al. study, and the Fielder et al. study, *fosB* was identified in all isolates (29, 47). While Bianco et al. (47) identified a relatively high number of isolates carrying a specific *mphB* allele (erythromycin resistance-conferring), we found a low prevalence of the *mphB* allele (2%), but higher prevalence of the *mph* allele (13%). Similarly to our findings, Bianco et al. detected *tetL* in just a single clinical isolate (47). Tetracycline resistance-conferring gene *tet45* was not identified among the clinical blood isolates studied in by Bianco et al. (47) but was abundant among isolates studied by Zhu et al. (48). Zhu et al. (48) identified 8 of the 17 isolates carrying *tet45* as B. cereus (*n* = 7) and B. thuringiensis (*n* = 1). In comparison, we identified a single *B. moscaicus* isolate that carried *tet45*.

### Comparison of antimicrobial phenotypes and genotypes revealed insufficient sensitivity and specificity for predicting antimicrobial resistance phenotypes based on the presence of ARGs.

Sensitivity and specificity were calculated for genes that were found in five isolates or more (*bla1*, *blaZ*, *mph*, and *rph*) detected by ABRIcate/MEGARes 2.0 and using broth microdilution results interpreted with CLSI M45 *Bacillus* spp. breakpoints for the corresponding antibiotics. *Bc* and *BcII* were excluded from the analysis due to their intrinsic resistance nature and the well characterized effect on β-lactam resistance (23, 43, 44). Further, all *van* genes identified were excluded from analysis as individual *van* genes are insufficient to confer phenotypic resistance to vancomycin (45, 46). ARGs that did not meet the abundance threshold (*n* ≥ 5) were excluded from the sensitivity and specificity analysis but were investigated to assess their effect on the MICs for their respective antibiotics. The single *blaTEM* isolate was phenotypically resistant to both ampicillin (>16 μg/mL) and penicillin (8 μg/mL). The isolate carrying *mphB* was susceptible to erythromycin (MIC <0.25 μg/mL). Two isolates carrying tetracycline resistance genes *tetL* and *tet45* were phenotypically resistant, both with MICs of >16 μg/mL. The isolates carrying *tet* genes had a higher MICs when compared to the MICs of isolates without *tet* genes. However, since only two isolates did not carry *tet* genes, this observation was not statistically assessed.

Sensitivity and specificity were calculated for *bla1* and *blaZ* genes' effect on ampicillin and penicillin resistance. *blaZ* had a higher sensitivity (56%) for prediction of both ampicillin and penicillin phenotypic resistance compared to *bla1* (29%) (Table 3). Of the two ampicillin susceptible isolates (MIC ≤ 0.25 μg/mL), each carried either a *bla1* or *blaZ*, resulting in a specificity of 50% for both of these ARGs. Since no isolates were susceptible to penicillin, *bla1* and *blaZ* showed a 100% specificity. Isolates that carried these genes had an average ampicillin MIC of >16 μg/mL and an average penicillin MIC of > 8 μg/mL, which was the same as the average MICs of the isolates that did not carry these genes.

**TABLE 3:** Sensitivity and specificity of antimicrobial resistance genes to predict phenotypic resistance

Antibiotic	AGR^*a*^	Sensitivity (%)^*b*^	Specificity (%)^*b*^
Ampicillin	*bla1*	28.92	50.00
	*blaZ*	56.63	50.00
Penicillin	*bla1*	29.41	100.00
	*blaZ*	56.47	100.00
Erythromycin	*mph*	0.00	98.52
Rifampicin	*rph*	0.00	93.42

a ARG, antimicrobial resistance gene.

b Sensitivity and specificity were calculated based on the broth microdilution results interpreted with CLSI M45 *Bacillus* spp. breakpoints. Only susceptible and resistant isolates, but not intermediate isolates, were included in the calculation.

There was no erythromycin resistance identified, yielding a sensitivity of 0% for *mph* (macrolide phosphotransferase) (Table 3). Further, of the 11 isolates carrying *mph*, only one isolate was susceptible (MIC was <0.25 μg/mL). This resulted in a high specificity of 99% (Table 3). The remaining 10 isolates carrying the *mph* gene were intermediate isolates. Average MICs were statistically compared between isolates with *mph* and without *mph* using a *t* test to investigate the effect of this gene on the MIC. The average MIC of *mph*-positive isolates (2.3 μg/mL) was significantly greater than the average MIC of isolates without the *mph* (0.4 μg/mL) (*P* < 0.05).

The five isolates carrying the *rph* gene were all susceptible to rifampicin and had an MIC <0.5 μg/mL, yielding a sensitivity of 0% and specificity of 93% (Table 3). The MICs of the isolates carrying *rph* (all <0.5 μg/mL) had a lower MIC when compared to isolates without the *rph* gene (average MIC was 0.88 μg/mL). Point mutations and potentially impaired function of *rpoB* due to premature stop codons were investigated for the seven rifampicin-resistant isolates. However, the point mutations identified in the resistant isolates’ *rpoB* sequence were also present in the susceptible isolates (Supplemental Material, Table S4). Further, no resistant isolates carried previously reported *rpoB* point mutations associated with rifampicin resistance (16).

Previous studies have not reported associations between antimicrobial resistance gene presence and phenotypic resistance of isolates from the B. cereus group species. Further, only two studies reported the presence of acquired antimicrobial resistance genes among studied isolates in combination with AST results (47, 48). Although the Bianco et al. study did not calculate the associations between presence of ARGs and phenotypic resistance, they did describe both (47). Bianco et al. identified β-lactamases in all isolates and further characterized all isolates as phenotypically resistant to penicillin G (47). Although they did not report the sensitivity and specificity, it is clear that the relationship between β-lactamase presence and phenotypic resistance was strong, which is similar to our findings (47). Bianco et al. identified a single isolate that carried the *tetL* gene and this isolate was identified as intermediately resistant to tetracycline. They had not identified any isolates as resistant. In contrast, all *tet-*positive isolates from our study were found to be phenotypically resistant (47). The gene *mphB* was identified in six isolates by Bianco et al., with 4 isolates intermediately resistant to erythromycin, suggestihg that macrolide phosphotransferases may have an effect on phenotypic resistance to macrolides, similar to our findings (47). Lastly, *Gly-vanR-M*, *Gly-vanZF-Pp*, and *vanR-B* genes were identified in 17, 15, and 2 out of 17 isolates in the Bianco et al. study; however, all isolates were phenotypically susceptible to vancomycin, as observed in our study (47). This is not surprising, given that an isolate must have all *vanM/vanB* glycopeptide resistance genes present in order to confer phenotypic resistance to vancomycin (45, 46). Multiple *van* genes that did not predict vancomycin resistance were identified also in our study. However, we did not detect *van* genes that have been previously associated with vancomycin resistance in *Enterococcus* (i.e., *vanA*, *vanB*, *vanC*) among the tested B. cereus isolates (46). Although we identified *rph* in our collection, it is clear it did not affect rifampicin resistance in the studied Bacillus cereus group isolates. Our findings, as well as Bianco et al. findings, show the need to further characterize the B. cereus group resistome and its effect on phenotypic resistance. Both of our studies investigated associations between the presence of acquired antimicrobial resistance genes and the phenotypic resistance and did not study the (over)expression of detected genes, which is required to detect phenotypic resistance. Further, some of the acquired genes must be highly conserved in order to result in a phenotypic resistance. Hence, the detection of ARG’s divergent variants may not be sufficient for expression of phenotypic resistance. Our findings show that more research is required to better understand the underlying mechanisms of the B. cereus group isolates’ antimicrobial resistance.

## MATERIALS AND METHODS

### Bacterial cultures.

A collection of 85 B. cereus group isolates that had been previously isolated from dairy foods and dairy-associated environments were included in this study (8, 33, 34). A list of all isolates used in this study and their corresponding metadata is available in Supplemental Material, Table S1. Isolates were resuscitated from cryo stocks preserved at −80°C by streaking a loopful of each cryo stock onto brain heart infusion (BHI) agar (BD Difco) and incubating a streaked plate for 15–18 h at 32°C. After completed incubation, a single isolated colony was sub-streaked onto a fresh BHI plate and incubated at the same conditions as outlined above. Subsequently, five isolated colonies were collected using a sterile loop and inoculated into 5 mL of BHI broth. Inoculated broths were incubated at 32°C for 15–18 h (49). The culture adjusted to 1–2 × 10^8^ was used for a disk diffusion assay described in the following section.

### Disk diffusion assay.

Disk diffusion assay was performed on each isolate by following the M100 Clinical and Laboratory Standards Institute (CLSI) guidelines (49). A total of nine antimicrobials applied on sterile disks were tested: ampicillin (10 μg/disk), ceftriaxone (30 μg/disk), ciprofloxacin (5 μg/disk), erythromycin (15 μg/disk), gentamicin (10 μg/disk), rifampicin (5 μg/disk), tetracycline (30 μg/disk), trimethoprim and sulfamethoxazole (1.25 + 23.75 μg/disk), and vancomycin (30 μg/disk) (49). Disks were produced by applying an antibiotic onto a sterile disk to achieve the defined mass or were purchased pre-made (HardyDisk AST, Hardy Diagnostics). Ampicillin, erythromycin, gentamicin, and rifampicin (Hardy Diagnostics) antibiotics were applied onto disks in the lab by preparing stock concentrations and aseptically applying 20 μl of each antibiotic onto a disk to achieve the target mass of antibiotic per disk. Disks were first let dry in a biosafety cabinet for 20 min and were then stored at −20°C until use. Ceftriaxone, ciprofloxacin, tetracycline, trimethoprim-sulfamethoxazole, and vancomycin antibiotics were purchased pre-applied on the disks and were stored at −20°C until use.

Bacterial cultures prepared as described in the previous section were streaked in a lawn onto Muller Hinton (MH) agar (Becton, Dickinson) using a sterile cotton swab (Medline Industries, Inc.) (49). Antimicrobial disks were placed onto the inoculated agar within 15 min of inoculation using sterile forceps (49). A maximum three disks were applied on each individual agar plate to avoid overlaps in zones of inhibition that could compromise the accuracy of zone measurement. Inoculated plates with antibiotic disks were incubated at 35°C for 16–20 h (49). Bacillus cereus type strain ATCC 14579 was included in each batch of tests for quality control. An uninoculated MH agar was also included in each batch of tests to control for medium sterility.

Inhibition zones were measured in millimeters using a ruler and interpreted using the guidelines and recommendations from CLSI M100 Staphylococcus spp. breakpoints (49) and guidelines were followed in cases when it was difficult to interpret zones of inhibition due to unclear edges (50). Examples include cases when some colonies grew within the zone of inhibition, when double zones of inhibition were observed, and when the antibiotic trimethoprim-sulfamethoxazole produced atypical and challenging to interpret inhibition zones (50).

### Broth microdilution assay.

Broth microdilution assays were performed on each isolate following the CLSI M45:ED3 guideline (32). A total of 18 antimicrobials were tested: ampicillin (0.12-16 μg/mL), ceftriaxone (8-64 μg/mL), ciprofloxacin (0.5-2 μg/mL), clindamycin (0.12-2 μg/mL), daptomycin (0.25-8 μg/mL), erythromycin (0.25-4 μg/mL), gatifloxacin (1-8 μg/mL), gentamicin (2-500 μg/mL), levofloxacin (0.25-8 μg/mL), linezolid (0.5-8 μg/mL), oxacillin + 2% NaCl (0.25-8 μg/mL), penicillin (0.06-8 μg/mL), quinupristin & dalfopristin (0.12-4 μg/mL), rifampicin (0.5-4 μg/mL), streptomycin (1000 μg/mL), tetracycline (2-16 μg/mL), trimethoprim & sulfamethoxazole (0.5/9.5-4/76 μg/mL), and vancomycin (1-64 μg/mL). The Sensititre GPN3F 96-well plates (Thermo Scientific) pre-loaded with antibiotics were used for broth microdilution testing. Bacillus cereus group isolates’ inocula were prepared from overnight cultures (see “Bacterial cultures” section) by first adjusting the culture concentration to 1–2 × 10^8^ CFU/mL using a spectrophotometer (Eppendorf BioPhotometer 6131). A previously established OD-CFU standard curve was used to estimate the CFU/mL based on the OD reading. The adjusted inoculum was then further diluted to achieve ∼5 × 10^5^ CFU/mL and dispensed into wells of a microtiter plate, 50 μL per well. Each plate included a positive control (no antibiotic added) and a negative control (just MH broth, no culture added). Inoculated plates were covered with a sealing tape provided with the Sensititre plates and incubated at 35°C for 18–24 h. To verify the inoculum concentration, dilutions of each inoculum were spread plated onto BHI agar and incubated at 30°C for 18–24 h. The plates were counted to ensure that the concentration of each inoculum loaded into the Sensititre plates were between 10^5^ and 10^6^ CFU/mL.

MICs were determined based on the guidelines and recommendations from CLSI. CLSI M07-A9 guideline was utilized in instances of unclear growth interpretations (51). This commonly included cases when a well had a very small amount of bacterial growth. In such cases, the bacterial pellets were measured in millimeters using a ruler and considered valid growth only if the pellet button was ≥ 2 mm (51). The MICs for antibiotics ampicillin, ciprofloxacin, clindamycin, erythromycin, gentamicin, levofloxacin, penicillin, rifampicin, tetracycline, trimethoprim-sulfamethoxazole, and vancomycin were interpreted with CLSI M45:ED3 *Bacillus* spp. guidelines (38). Linezolid breakpoints were not defined for *Bacillus* spp. in CLSI M45; hence, EUCAST v12.0 *Bacillus* spp. breakpoints were applied (21). The remaining antibiotics, ceftriaxone, gatifloxacin, oxacillin +2%NaCl, quinupristin-dalfopristin, daptomycin, and streptomycin did not have clinical breakpoints defined for *Bacillus* spp. in either CLSI M45:ED3 or EUCAST v12.0. ECOFFinder of EUCAST was attempted to be used to determine ecological cutoff values (ECVs), however our data distributions did not meet the criteria for determination of EUCAST ECVs (40, 41).

### Comparative analysis of disk diffusion and broth microdilution results.

The results for nine antibiotics (i.e., ampicillin, ceftriaxone, ciprofloxacin, erythromycin, gentamicin, rifampicin, tetracycline, trimethoprim-sulfamethoxazole, and vancomycin) analyzed with disk diffusion and broth microdilution assays were compared to investigate the correlation between zones of inhibition and MICs. This analysis was visualized via scatterplots, plotted in R and R Studio v4.0.4 using packages base v4.0.4, graphics v4.0.4, ggplot v3.3.3, grDevices v4.0.4, methods v4.0.4, stats v4.0.4, and scales v1.1., and R^2^ was calculated to quantify the correlations between the results obtained by these two methods (52). Further, minor, major, and very major errors, and categorical agreement were calculated as outlined by Patel et al. (53). Errors were calculated to assess the reliability of disk diffusion results interpreted with CLSI M100 Staphylococcus spp. breakpoints compared to the results obtained using the gold standard method, broth microdilution, interpreted with CLSI M45 *Bacillus* spp. breakpoints. This analysis was applied to the seven antibiotics (i.e., ampicillin, ciprofloxacin, erythromycin, gentamicin, rifampicin, tetracycline, and trimethoprim-sulfamethoxazole) that had defined breakpoints in CLSI M100 Staphylococcus spp. and CLSI M45 *Bacillus* spp. guidelines.

### Genotyping, phylogenetic analysis, and detection of antimicrobial resistance genes using whole-genome sequencing.

Sequencing reads for the 85 Bacillus cereus group isolates that had been previously sequenced were obtained from the NCBI SRA database (8, 33, 34). Sequence accession numbers are listed in the Supplemental Material, Table S1. The quality of sequences was examined with FASTQC v0.11.8 using default settings (54). Poor quality bases and adaptors were removed with Trimmomatic v0.39 using default settings and Nextera PE adapter sequences (55). Trimmed sequences were then assembled using SPAdes v3.15 with the kmer sizes of 99 and 127 and the --careful option (56). Assembled genomes were checked for quality using QUAST v5.0.2 using the default parameters (57).

Draft genomes and processed reads were analyzed using ABRIcate v1.01 and ARIBA v2.14.6, respectively, to detect the presence of acquired resistance genes (58, 59). MEGARes 2.0 and ResFinder 4.1 databases were used with both programs. Gene presence was determined with each program using their respective default ≥ 90% percent coverage and identity. Point mutations in *rpoB* were analyzed through creating a consensus alignment of all 85 isolates and tracking amino acid changes.

BTyper3 v3.3.1 was used for taxonomic identification, genotyping (i.e., *panC*, *rpoB*, MLST typing) and identification of virulence genes using default settings (60, 61). PROKKA v1.14.6 was used to annotate genomes and produce GFF files that were then used for the analysis of pangenome with Roary v3.11.2 (62, 63). The core genome alignment produced by Roary was used for phylogenetic inference with RAxML v8.2.12 (63, 64). RAxML was run with 1,000 bootstrap repetitions and the tree with the highest likelihood was used for visualization of phylogenetic relationships among isolates. The code for the bioinformatic analysis workflow outlined above is available at https://github.com/EmmaMills101/Bacillus-Cereus-Workflow.

### Data visualization and statistical analyses.

Distributions of disk diffusion zones of inhibition and broth microdilution MICs for each antibiotic were plotted in R and R Studio v4.0.4 using packages base v4.0.4, graphics v4.0.4, ggplot v3.3.3, grDevices v4.0.4, methods v4.0.4, and stats v4.0.4 (58). Phylogenetic tree, sequence types, virulence genes, antibiotic susceptibility testing results, and ARGs were visualized using iTOL v5 (65). The average MICs of select antibiotics were compared using a *t* test with 5% significance. The sensitivity and specificity for predicting phenotypic antibiotic resistance based on detected ARGs were calculated using the guidelines outlined in Trevethan, 2017 (66).
