# Supplementary material for: Comparative Analysis of Bacillus cereus Group Isolates' Resistance Using Disk Diffusion and Broth Microdilution and the Correlation between Antimicrobial Resistance Phenotypes and Genotypes
Source: Appl Environ Microbiol. 2022 Mar 22;88(6):e02302-21. doi: 10.1128/aem.02302-21 (PMC8939351; doi:10.1128/aem.02302-21)
Supplement: Supplemental file 1 — Tables S1 to S4 and Fig. S1 to S3. Download aem.02302-21-s0001.pdf, PDF file, 1.6 MB [file aem.02302-21-s0001.pdf]

SUPPLEMENTAL MATERIAL

Comparative analysis of *Bacillus cereus* group isolates' resistance using disk diffusion and broth microdilution and the correlation between antimicrobial resistance phenotypes and genotypes

Emma Mills[a], Erin Sullivan[a], Jasna Kovac[a#]

[a]Department of Food Science, The Pennsylvania State University, The Pennsylvania State University, University Park, Pennsylvania, United States

[#]Corresponding author: Jasna Kovac, jzk303@psu.edu

SUPPLEMENTAL TABLET S1: Metadata for 85 *B. cereus* group isolates tested in this study.

Isolate	SRA Accession Number	Isolate Source[a]	Collection Year[a]	State of Isolation[a]	N50 (Kbp)	GC (%)	Length (Mbp)	Number of Contigs ≥ 1000 bp	Taxon ID	*panC* Clade	MLST	*nhe Genes*	*hbl Genes*	*cytK*	Bt-encoding Genes
FSL F4-0079	SRR4661787	Pasteurized Milk	2002	NY	215.2	35.55	5.3	53	*B. mycoides*	*VI mycoides/paramycoides*	1091	*nheA, nheB, nheC*	*hblA, hblB, hblC, hblD*		
FSL H7-0683	SRR4064645	Pasteurized Milk	2005	NY	118.7	35.22	6	116	*B. mycoides*	*VI mycoides/paramycoides*	222	*nheA, nheB, nheC*	*hblA, hblB, hblC, hblD*		
FSL H7-0909	SRR4661785	Raw Milk	2005	NY	166.9	35.2	5.9	84	*B. mycoides*	*VI mycoides/paramycoides*	222	*nheA, nheB, nheC*	*hblA, hblB, hblC, hblD*		
FSL H7-0676	SRR4661786	Raw Milk	2005	NY	58.5	35.34	5.8	248	*B. mycoides biovar Thuringiensis, B. Thuringiensis*	*VI mycoides/paramycoides*	325	*nheA, nheB, nheC*	*hblA, hblB, hblC, hblD*		*5Cry19Ba1, Cry19Ca1, Cry27Aa1, Cry56Aa4, Cry70Bb1*
FSL H7-0926	SRR2541537	Pasteurized Milk	2005	NY	313.4	35.41	5.4	34	*B. mycoides*	*VI mycoides/paramycoides*	667	*nheA, nheB, nheC*	*hblA, hblC, hblD*		
FSL H8-0063	SRR4661788	Raw Milk	2005	NY	133.8	35.26	5.6	126	*B. mycoides*	*VI mycoides/paramycoides*	721	*nheA, nheB, nheC*	*hblA, hblB, hblC, hblD*		
FSL H7-0344	SRR5185037	Pasteurized Milk	2005	NY	432	35.46	5.3	27	*B. mosaicus*	*II mosaicus/luti*	1097	*nheA, nheB, nheC*			
FSL H7-0611	SRR4064644	Pasteurized Milk	2005	NY	116.9	35.51	5.3	109	*B. mosaicus*	*II mosaicus/luti*	1097	*nheA, nheB, nheC*			
FSL H7-0353	SRR3458441	Pasteurized Milk	2005	NY	406.2	35.12	5.6	36	*B. mosaicus*	*II mosaicus/luti*	1272	*nheA, nheB, nheC*	*hblA, hblB, hblC, hblD*		
FSL H7-0444	SRR5185036	Pasteurized Milk	2005	NY	382.9	35.12	5.6	38	*B. mosaicus*	*II mosaicus/luti*	1272	*nheA, nheB, nheC*	*hblA, hblB, hblC, hblD*		
FSL H8-0032	SRR3458442	Pasteurized Milk	2005	NY	303.2	35.12	5.6	32	*B. mosaicus*	*II mosaicus/luti*	1272	*nheA, nheB, nheC*	*hblA, hblB, hblC, hblD*		
FSL H8-0049	SRR5185035	Pasteurized Milk	2005	NY	428.5	35.25	5.3	31	*B. mosaicus*	*II mosaicus/luti*	1272	*nheA, nheB, nheC*	*hblA, hblB, hblC, hblD*		
FSL R5-0585	SRR5185019	Dairy	2006	FL	186.6	35	5.6	105	*B. cereus s.s.*	*IV cereus sensu stricto*	1143	*nheA, nheB, nheC*	*hblA, hblB, hblC, hblD*	cytK-2	
FSL R5-0708	SRR5189062	Dairy	2006	GA	207.4	35.45	5.2	63	*B. mycoides*	*VI mycoides/paramycoides*	1144	*nheA, nheB, nheC*	*hblA, hblB, hblC, hblD*		
FSL R5-0184	SRR5185020	Dairy	2006	MI	136.2	34.97	6	124	*B. cereus s.s.*	*IV cereus sensu stricto*	927	*nheA, nheB, nheC*	*hblA, hblB, hblC, hblD*	cytK-2	

FSL H8-0534	SRR4661789	Soil	2006	NY	121.2	35.48	5.9	123	*B. pseudomycoides*	*I pseudomycoides*	83	*nheA, nheB, nheC*	*hblA, hblB, hblC, hblD*		
FSL H8-0488	SRR2541602	Water	2006	NY	463.8	34.95	5.9	45	*B. toyonensis*	*V toyonensis*	111	*nheA, nheB, nheC*	*hblA, hblB, hblC, hblD*		
FSL R7-0047	SRR5185014	Dairy	2006	NY	71.6	35.27	5.7	229	*B. mycoides*	*VI mycoides/paramycoides*	222	*nheA, nheB, nheC*	*hblA, hblB, hblC, hblD*		
FSL H8-0482	SRR2541601	Soil	2006	NY	379.1	34.86	6	57	*B. toyonensis*	*V toyonensis*	223	*nheA, nheB, nheC*	*hblA, hblB, hblC, hblD*		
FSL H8-0492	SRR4661790	Raw Milk	2006	NY	271.8	35.46	5.5	44	*B. mycoides*	*VI mycoides/paramycoides*	1098	*nheA, nheB, nheC*	*hblC, hblD*		
FSL R5-0811	SRR5189061	Dairy	2006	NY	107.3	35.13	5.7	114	*B. cereus s.s.*	*IV cereus sensu stricto*	1099	*nheA, nheB, nheC*	*hblA, hblB, hblC, hblD*	cytK-2	
FSL R5-0832	SRR5185017	Dairy	2006	NY	147.5	35.11	5.7	69	*B. cereus s.s.*	*IV cereus sensu stricto*	1099	*nheA, nheB, nheC*	*hblA, hblB, hblC, hblD*	cytK-2	
FSL R5-0859	SRR5185016	Dairy	2006	NY	116.1	35.11	5.7	88	*B. cereus s.s.*	*IV cereus sensu stricto*	1099	*nheA, nheB, nheC*	*hblA, hblB, hblC, hblD*	cytK-2	
FSL R7-0117	SRR5185013	Dairy	2006	NY	147.5	35.09	5.7	96	*B. cereus s.s.*	*IV cereus sensu stricto*	1099	*nheA, nheB, nheC*	*hblA, hblB, hblC, hblD*	cytK-2	
FSL R7-0282	SRR5185012	Dairy	2006	NY	134.7	35.13	5.7	82	*B. cereus s.s.*	*IV cereus sensu stricto*	1099	*nheA, nheB, nheC*	*hblA, hblB, hblC, hblD*	cytK-2	
FSL H8-0485	SRR5189059	Soil	2006	NY	178.1	35.1	6.3	95	*B. mycoides biovar Thuringiensis, B. Thuringiensis*	*VI mycoides/paramycoides*	1142	*nheA, nheB, nheC*	*hblC, hblD*		*3Cry22Ba1, Cry32Qa1, Cry32Ya1*
FSL H8-0481	SRR4661784	Soil	2006	NY	59.3	35.58	5.3	221	*B. pseudomycoides*	*I pseudomycoides*	1346	*nheC*	*hblA, hblB, hblC, hblD*		
FSL H8-0545	SRR4064642	Water	2006	NY	147	35.32	5.3	238	*B. mycoides*	*VI mycoides/paramycoides*	2764	*nheC*	*hblA, hblB, hblC, hblD*		
FSL R5-0920	SRR5185015	Dairy	2006	NY	210.8	35.55	5.3	55	*B. mycoides*	*VI mycoides/paramycoides*	2766	*nheA, nheB, nheC*	*hblC, hblD*		
FSL R5-0594	SRR5185018	Dairy	2006	TX	117.3	35.28	5.9	83	*B. mosaicus*	*II mosaicus/luti*	1096	*nheA, nheB, nheC*	*hblA, hblB, hblC, hblD*	cytK-2	
FSL P2-0558	SRR3458448	Food in process	2009	NY	727.5	35.11	5.6	41	*B. mosaicus*	*II mosaicus/luti*	644	*nheA, nheB, nheC*	*hblA, hblB, hblC, hblD*		
FSL P2-0235	SRR5185023	Food in process	2009	NY	81.4	35.08	5.6	123	*B. cereus s.s.*	*IV cereus sensu stricto*	1099	*nheA, nheB, nheC*	*hblA, hblB, hblC, hblD*	cytK-2	
FSL P2-0415	SRR3458447	Food in process	2009	NY	219.9	35.19	5.6	44	*B. mosaicus*	*II mosaicus/luti*	1268	*nheA, nheB, nheC*	*hblA, hblB, hblC, hblD*	cytK-2	
FSL P2-0021	SRR5185024	Food in process	2009	NY	229.8	35.43	5.1	71	*B. mycoides*	*VI mycoides/paramycoides*	2764	*nheA, nheB, nheC*	*hblA, hblB, hblC, hblD*		
FSL P4-0488	SRR5185021	Food in process	2010	NY	42.1	35.2	5.9	341	*B. mycoides*	*VI mycoides/paramycoides*	222	*nheA, nheB, nheC*	*hblA, hblB, hblC, hblD*		

FSL W7-1108	SRR5189056	Food in process	2010	NY	221.1	35.09	6.1	95	*B. mycoides*	*VI mycoides/paramycoides*	673	*nheA, nheB, nheC*	*hblA, hblB, hblC, hblD*		
FSL W7-1101	SRR5185011	Food in process	2010	NY	300.4	34.84	6.3	54	*B. cereus s.s.*	*IV cereus sensu stricto*	1100	*nheA, nheB, nheC*	*hblA, hblB, hblC, hblD*	cytK-2	
FSL P4-0569	SRR3458449	Food in process	2010	NY	737.9	35.24	5.4	28	*B. mosaicus*	*II mosaicus/luti*	1266	*nheA, nheB, nheC*	*hblC, hblD*	cytK-2	
FSL P4-0260	SRR5185022	Food in process	2010	NY	99.7	35.36	5.5	134	*B. mycoides*	*VI mycoides/paramycoides*	2766	*nheA, nheB, nheC*	*hblA, hblB, hblC, hblD*		
FSL W7-1334	SRR5185009	Food in process	2011	MN	180.3	35.05	5.8	119	*B. cereus s.s.*	*IV cereus sensu stricto*	265	*nheA, nheB, nheC*	*hblA, hblB, hblC, hblD*		
FSL W7-1328	SRR5185010	Food in process	2011	MN	27.5	35.38	6	404	*B. mosaicus*	*II mosaicus/luti*	1093	*nheA, nheB, nheC*		cytK-2	
FSL M7-1090	SRR5185029	Raw Milk	2011	NY	78.8	35.32	5.6	181	*B. mycoides*	*VI mycoides/paramycoides*	196	*nheA, nheB, nheC*	*hblA, hblB, hblC, hblD*		
FSL M7-0109	SRR5189057	Raw Milk	2011	NY	560.9	35.13	5.8	36	*B. mycoides*	*VI mycoides/paramycoides*	410	*nheA, nheB, nheC*	*hblA, hblB, hblC, hblD*		
FSL M7-0322	SRR5185032	Raw Milk	2011	NY	216.2	35.36	5.6	64	*B. mycoides*	*VI mycoides/paramycoides*	410	*nheA, nheB, nheC*	*hblA, hblB, hblC, hblD*		
FSL M7-0053	SRR4661791	Raw Milk	2011	NY	428.5	35.4	5.3	48	*B. mycoides*	*VI mycoides/paramycoides*	414	*nheA, nheB, nheC*	*hblA, hblB, hblC, hblD*		
FSL M7-1006	SRR5185030	Raw Milk	2011	NY	74.8	35.25	5.9	176	*B. mycoides*	*VI mycoides/paramycoides*	625	*nheA, nheB, nheC*	*hblA, hblB, hblC, hblD*		
FSL M7-1219	SRR5189064	Raw Milk	2011	NY	78.2	35.25	5.7	172	*B. mycoides*	*VI mycoides/paramycoides*	1092	*nheA, nheB, nheC*	*hblA, hblB, hblC, hblD*		
FSL M7-0690	SRR5185031	Raw Milk	2011	NY	213.1	35.18	5.5	52	*B. mosaicus*	*II mosaicus/luti*	1267	*nheA, nheB, nheC*	*hblA, hblB, hblC, hblD*		
FSL M7-0938	SRR3458445	Raw Milk	2011	NY	345.5	35.21	5.4	35	*B. mosaicus*	*II mosaicus/luti*	1269	*nheA, nheB, nheC*	*hblA, hblB, hblC, hblD*		
FSL M7-1251	SRR3458446	Raw Milk	2011	NY	255	35.2	5.4	47	*B. mosaicus*	*II mosaicus/luti*	1270	*nheA, nheB, nheC*	*hblA, hblB, hblC, hblD*	cytK-2	
FSL M7-0044	SRR3458444	Raw Milk	2011	NY	363.7	35.21	5.4	29	*B. mosaicus*	*II mosaicus/luti*	1271	*nheA, nheB, nheC*	*hblA, hblB, hblC, hblD*	cytK-2	
FSL W8-0824	SRR2541693	Food in process	2012	NY	190.5	35.11	5.5	101	*B. cereus s.s.*	*IV cereus sensu stricto*	24	*nheA, nheB, nheC*	*hblA, hblB, hblC, hblD*	cytK-2	
FSL W8-0050	SRR2541641	Food in process	2012	NY	223.9	35.25	5.5	64	*B. mosaicus*	*III mosaicus*	32	*nheA, nheB, nheC*		cytK-2	
FSL K6-0073	SRR2541607	Raw Milk	2012	NY	91.4	34.49	6.9	177	*B. cereus s.s. biovar Thuringiensis, B. Thuringiensis*	*IV cereus sensu stricto*	33	*nheA, nheB, nheC*	*hblA, hblB, hblC, hblD*	cytK-2	*3Cry1Ia24, Vip1Ad1, Vip2Ad1*
FSL K6-0220	SRR4064641	Raw Milk	2012	NY	33.5	35.6	5.8	404	*B. pseudomycoides*	*I pseudomycoides*	83	*nheA, nheB, nheC*	*hblA, hblB, hblC, hblD*		

FSL M8-0139	SRR5185026	Raw Milk	2012	NY	24.4	35.22	5.7	379	B. cereus s.s. biovar Thuringiensis, B. Thuringiensis	IV cereus sensu stricto	138	nheA, nheB, nheC		cytK-2	2Cry34Ac1, Cry35Ac1
FSL M8-0214	SRR5185025	Raw Milk	2012	NY	159.9	35.05	5.9	117	B. toyonensis	V toyonensis	223	nheA, nheB, nheC	hblA, hblB, hblC, hblD		
FSL K6-0268	SRR5185034	Raw Milk	2012	NY	46.5	35.03	6.5	351	B. cereus s.s.	IV cereus sensu stricto	230	nheA, nheB, nheC	hblA, hblB, hblC, hblD	cytK-2	
FSL M7-1472	SRR5185027	Raw Milk	2012	NY	34.2	35.31	5.4	371	B. mosaicus	II mosaicus/luti	564	nheA, nheB, nheC	hblA, hblB, hblC, hblD		
FSL M8-0091	SRR5189063	Raw Milk	2012	NY	441.4	35.32	5.5	36	B. mosaicus	II mosaicus/luti	564	nheA, nheB, nheC	hblA, hblB, hblC, hblD		
FSL W8-0767	SRR2541718	Food in process	2012	NY	12.5	35.01	5.6	814	B. cereus s.s.	IV cereus sensu stricto	787	nheA, nheB, nheC	hblA, hblB, hblC, hblD	cytK-2	
FSL W8-0520	SRR2541680	Food in process	2012	NY	79.4	35.4	5.4	189	B. mosaicus	III mosaicus	1032	nheA, nheB, nheC		cytK-2	
FSL W8-0523	SRR2541686	Food in process	2012	NY	238.8	35.31	5.3	58	B. mosaicus	III mosaicus	1032	nheA, nheB, nheC		cytK-2	
FSL W8-0275	SRR2541668	Food in process	2012	NY	148	35.1	5.6	95	B. mosaicus	III mosaicus	1050	nheA, nheB, nheC		cytK-2	
FSL K6-0069	SRR2541606	Raw Milk	2012	NY	135.8	35.35	5.6	66	B. mosaicus	II mosaicus/luti	1080	nheA, nheB, nheC		cytK-2	
FSL W8-0169	SRR2541651	Food ingredient	2012	NY	395.9	35.21	5.3	40	B. mosaicus	II mosaicus/luti	1081	nheA, nheB, nheC	hblA, hblB, hblC, hblD	cytK-2	
FSL W8-0483	SRR2541674	Food in process	2012	NY	145.8	35.16	5.4	82	B. mosaicus	III mosaicus	1082	nheA, nheB, nheC			
FSL W8-0268	SRR2541662	Food in process	2012	NY	293.7	35.05	5.8	48	B. cereus s.s.	IV cereus sensu stricto	1083	nheA, nheB, nheC	hblA, hblB, hblC, hblD	cytK-2	
FSL W8-0003	SRR2541640	Food in process	2012	NY	138.6	35.09	5.8	106	B. mosaicus	III mosaicus	1084	nheA, nheB, nheC			
FSL M8-0117	SRR2541639	Raw Milk	2012	NY	140.5	35.07	5.9	126	B. mosaicus biovar Thuringiensis, B. Thuringiensis	II mosaicus/luti	1085	nheA, nheB, nheC	hblA, hblB, hblC, hblD	cytK-2	3Cry21Aa3, Cry21Ga1, Cry5Ac1
FSL K6-0067	SRR2541605	Raw Milk	2012	NY	48.6	35.17	5.9	283	B. mosaicus	II mosaicus/luti	1086	nheA, nheB, nheC		cytK-2	
FSL K6-0043	SRR2541604	Raw Milk	2012	NY	794.3	34.93	5.7	28	B. cereus s.s.	IV cereus sensu stricto	1087	nheA, nheB, nheC	hblA, hblB, hblC, hblD	cytK-2	
FSL W8-0640	SRR2541688	Food in process	2012	NY	177.3	35.01	5.9	121	B. cereus s.s.	IV cereus sensu stricto	1089	nheA, nheB, nheC	hblA, hblB, hblC, hblD	cytK-2	
FSL K6-0267	SRR2541613	Raw Milk	2012	NY	269.7	35.28	5.7	65	B. mycoides	VI mycoides/paramycoides	1090	nheA, nheB, nheC	hblC, hblD		

FSL J3-0113	SRR3458443	Pasteurized Milk	2012	NY	1130.7	35.08	5.6	21	*B. mosaicus*	*II mosaicus/luti*	1094	*nheA, nheB, nheC*	*hblC, hblD*		
FSL J3-0123	SRR5189058	Pasteurized Milk	2012	NY	298.1	35.39	5.1	44	*B. mycoides*	*VI mycoides/paramycoides*	1095	*nheA, nheB, nheC*	*hblA, hblB, hblC, hblD*		
FSL M7-1345	SRR5185028	Raw Milk	2012	NY	128.4	35.6	5	63	*B. mycoides*	*VI mycoides/paramycoides*	1095	*nheA, nheB, nheC*	*hblA, hblB, hblC, hblD*		
FSL K6-0042	SRR5185033	Raw Milk	2012	NY	147	35.46	5.3	97	*B. pseudomycoides*	*I pseudomycoides*	1346	*nheC*	*hblA, hblB, hblC, hblD*		
FSL K6-0040	SRR2541603	Raw Milk	2012	NY	194.2	34.73	6.1	110	*B. cereus s.s.*	*IV cereus sensu stricto*	2034	*nheA, nheB, nheC*	*hblA, hblB, hblC, hblD*	cytK-2	
FSL W8-0932	SRR2541715	Food in process	2013	NY	84.5	35.2	5.5	127	*B. mosaicus*	*III mosaicus*	365	*nheA, nheB, nheC*			
FSL K6-1030	SRR4661783	Condensed Milk	2013	PA	38.2	35.18	6.6	349	*B. cereus s.s.*	*IV cereus sensu stricto*	1968	*nheA, nheB, nheC*	*hblA, hblB, hblC, hblD*	cytK-2	
FSL K6-3163	SRR4661792	Raw Milk	2014	MD	26.3	35.3	5.3	417	*B. cereus s.s.*	*IV cereus sensu stricto*	146	*nheA, nheB, nheC*	*hblA, hblB, hblC, hblD*	cytK-2	
FSL E2-0214	SRR4661781	Pasteurized Milk	2014	NY	207	35.43	5.4	73	*B. mycoides*	*VI mycoides/paramycoides*	410	*nheA, nheB, nheC*	*hblA, hblB, hblC, hblD*		
FSL K6-1142	SRR4661782	Teat-swab	2014	NY	72.7	35.55	5.3	165	*B. pseudomycoides*	*I pseudomycoides*	1346	*nheC*	*hblA, hblB, hblC, hblD*		
FSL M8-0473	SRR5189060	ND	ND	ND	91.6	35.2	5.4	138	*B. cereus s.s.*	*IV cereus sensu stricto*	4	*nheA, nheB, nheC*	*hblA, hblB, hblC, hblD*	cytK-2	

[a] ND, not determined or unknown.

SUPPLEMENTAL TABLE S2: Antimicrobial susceptibility testing results for 85 *B. cereus* group isolates.

	Ampicillin		Ceftriaxone		Clindamycin	Ciprofloxacin		Daptomycin	Erythromycin		Gatifloxacin	Gentamicin		Levofloxacin	Linezolid	Oxacillin + 2%NaCl	Penicillin	Quinupristin & Dalfopristin	Rifampicin		Streptomycin	Tetracycline		Trimethoprim / Sulfamethoxazole		Vancomycin	
Isolate	Inhibition Zone (mm)[a]	MIC[c]	Inhibition Zone (mm)[b]	MIC[b]	MIC[c]	Inhibition Zone (mm)[a]	MIC[c]	MIC[b]	Inhibition Zone (mm)[a]	MIC[c]	MIC[b]	Inhibition Zone (mm)[a]	MIC[c]	MIC[c]	MIC[d]	MIC[b]	MIC[c]	MIC[b]	Inhibition Zone (mm)[a]	MIC[c]	MIC[b]	Inhibition Zone (mm)[a]	MIC[c]	Inhibition Zone (mm)[a]	MIC[c]	Inhibition Zone (mm)[b]	MIC[c]
FSL H8-0488	0	>16	7	64	2	17	<0.5	8	12	4	1	25	<2	0.25	8	8	8	4	10	>4	<1000	13	<2	0	>4/76	8	>128
FSL W8-0640	0	>16	0	>64	1	22	<0.5	8	25	<0.25	1	26	<2	0.25	1	8	8	1	11	<0.5	<1000	13	4	0	>4/76	11	2
FSL R5-0585	9	>16	0	>64	1	22	<0.5	8	31	<0.25	1	31	<2	0.25	1	8	8	2	10	<0.5	<1000	18	<2	0	>4/76	11	2
FSL R5-0811	0	>16	0	>64	0.5	19	<0.5	8	24	<0.25	1	25	<2	0.25	1	8	8	1	14	<0.5	<1000	14	<2	0	>4/76	12	2
FSL W8-0523	30	>16	18	32	0.5	30	2	8	37	2	1	39	16	2	2	8	8	2	25	>4	<1000	25	<2	0	>4/76	12	2
FSL W8-0767	0	>16	0	>64	1	18	<0.5	8	27	<0.25	1	27	4	0.25	1	8	8	2	11	<0.5	<1000	15	4	0	>4/76	13	2
FSL H8-0482	0	>16	0	>64	0.25	20	<0.5	8	13	2	1	26	<2	0.5	1	8	8	2	11	<0.5	<1000	16	<2	0	>4/76	13	4
FSL H8-0492	10	>16	9	64	0.12	22	<0.5	8	23	<0.25	1	20	<2	0.25	0.5	8	8	2	17	<0.5	<1000	19	<2	0	>4/76	13	<1
FSL K6-0073	0	>16	10	>64	0.5	33	<0.5	8	36	<0.25	1	37	<2	0.25	1	8	8	1	24	<0.5	<1000	25	<2	0	>4/76	13	2
FSL M8-0091	14	>16	11	32	0.25	21	<0.5	8	25	<0.25	1	25	<2	0.25	1	8	8	1	15	<0.5	<1000	14	<2	0	>4/76	14	2
FSL K6-0043	10	>16	0	>64	1	23	<0.5	8	29	<0.25	1	30	4	0.25	1	8	8	2	10	<0.5	<1000	15	4	0	>4/76	14	2
FSL M7-1251	0	>16	0	>64	0.5	21	<0.5	8	23	<0.25	1	26	<2	0.25	1	8	8	1	16	<0.5	<1000	15	<2	0	>4/76	14	2
FSL M8-0139	8	>16	0	>64	0.5	20	<0.5	8	28	<0.25	1	30	4	0.25	1	8	8	2	26	<0.5	<1000	16	<2	0	>4/76	14	2
FSL W8-0169	9	>16	0	>64	0.5	18	1	8	24	1	1	25	16	2	1	8	8	1	13	4	<1000	17	<2	0	>4/76	14	2
FSL H8-0032	0	>16	0	>64	0.25	20	<0.5	4	27	<0.25	1	25	<2	0.25	1	8	8	2	15	<0.5	<1000	17	<2	0	>4/76	14	2
FSL M7-1090	11	>16	11	>64	0.5	25	<0.5	8	26	<0.25	1	29	<2	0.25	1	8	8	2	17	<0.5	<1000	20	<2	0	>4/76	14	2

FSL K6-3163	20	0.25	13	16	0.12	21	<0.5	4	29	<0.25	1	21	<2	0.25	0.5	0.25	1	0.5	27	<0.5	<1000	27	<2	0	>4/76	14	<1
FSL K6-0042	25	2	12	64	2	21	<0.5	8	18	<0.25	1	35	<2	0.25	0.5	8	1	2	23	<0.5	<1000	0	<2	0	>4/76	15	<1
FSL H8-0063	20	>16	13	>64	0.12	20	<0.5	8	30	<0.25	1	35	<2	0.25	0.5	8	8	0.5	18	<0.5	<1000	8	<2	0	>4/76	15	<1
FSL H8-0481	23	2	14	32	2	23	<0.5	8	31	<0.25	1	25	<2	0.25	1	0.25	2	2	20	<0.5	<1000	9	<2	0	>4/76	15	<1
FSL W8-0268	13	>16	9	32	0.5	18	<0.5	8	22	<0.25	1	26	<2	0.5	1	8	8	1	12	<0.5	<1000	10	4	0	>4/76	15	<1
FSL M7-0044	11	>16	0	>64	0.5	21	<0.5	8	24	<0.25	1	25	<2	0.25	1	8	8	1	14	<0.5	<1000	15	<2	0	>4/76	15	2
FSL H7-0444	0	>16	0	>64	0.25	13	<0.5	8	24	<0.25	1	28	<2	0.25	1	8	8	1	14	<0.5	<1000	15	<2	0	>4/76	15	2
FSL M7-0053	12	>16	8	64	0.25	17	<0.5	8	25	<0.25	1	26	<2	0.25	1	8	8	2	15	<0.5	<1000	15	<2	0	>4/76	15	<1
FSL K6-0067	24	<0.12	0	<8	1	21	<0.5	2	27	<0.25	1	26	<2	0.25	1	0.25	2	1	15	<0.5	<1000	15	<2	0	>4/76	15	<1
FSL W8-0050	0	>16	0	>64	0.5	18	<0.5	8	27	<0.25	1	25	4	0.25	1	8	8	1	13	<0.5	<1000	17	<2	0	>4/76	15	2
FSL R5-0594	14	>16	0	>64	1	24	<0.5	8	24	<0.25	1	31	<2	0.25	2	8	8	1	15	<0.5	<1000	18	<2	0	>4/76	15	2
FSL R7-0117	16	>16	8	>64	1	22	1	8	26	<0.25	1	30	4	0.5	1	8	8	1	18	1	<1000	18	<2	0	>4/76	15	2
FSL W8-0483	12	>16	9	32	1	19	<0.5	8	15	2	1	29	<2	0.25	1	8	8	2	12	<0.5	<1000	19	<2	0	>4/76	15	4
FSL W8-0932	0	>16	0	>64	1	21	<0.5	8	16	2	1	27	4	0.25	1	8	8	2	14	<0.5	<1000	19	<2	0	>4/76	15	2
FSL R5-0832	12	>16	0	>64	1	21	<0.5	8	27	0.5	1	29	4	2	1	8	8	1	17	1	<1000	19	<2	0	>4/76	15	2
FSL R5-0184	13	>16	9	>64	1	22	<0.5	8	28	<0.25	1	30	<2	0.25	1	8	8	1	13	<0.5	<1000	17	<2	7	>4/76	15	2
FSL J3-0113	10	>16	8	64	1	20	<0.5	8	19	4	1	28	<2	0.25	1	8	8	2	15	<0.5	<1000	15	<2	9	>4/76	15	2
FSL K6-1142	22	1	14	16	2	29	<0.5	4	33	<0.25	1	32	<2	0.25	1	0.25	1	1	25	<0.5	<1000	29	<2	12	>4/76	15	<1
FSL H7-0676	15	>16	10	>64	0.12	25	<0.5	8	27	<0.25	1	27	<2	0.25	1	0.25	8	2	18	<0.5	<1000	20	<2	15	>4/76	15	<1

FSL H7-0909	0	>16	10	>64	0.12	25	<0.5	2	21	<0.25	1	34	<2	0.25	1	8	8	2	17	<0.5	<1000	15	<2	0	>4/76	16	<1
FSL M7-1006	14	>16	9	>64	0.5	24	<0.5	8	24	<0.25	1	27	<2	0.25	0.5	8	8	2	20	<0.5	<1000	19	<2	0	>4/76	16	<1
FSL P2-0415	28	>16	9	>64	1	26	<0.5	8	35	<0.25	1	30	4	0.25	2	8	8	2	19	<0.5	<1000	22	<2	0	>4/76	16	2
FSL F4-0079	28	>16	15	64	0.12	28	<0.5	8	35	0.5	1	38	<2	0.25	1	8	8	1	27	<0.5	<1000	24	<2	0	>4/76	16	<1
FSL H7-0611	29	>16	12	32	0.25	24	<0.5	8	21	<0.25	1	28	4	0.25	1	8	8	1	14	<0.5	<1000	20	<2	17	>4/76	16	2
FSL P4-0488	21	>16	12	64	0.25	21	<0.5	8	36	<0.25	1	35	<2	0.25	1	8	8	2	19	<0.5	<1000	24	<2	21	>4/76	16	<1
FSL M8-0117	11	>16	11	>64	2	23	1	8	25	4	1	30	512	2	8	8	8	4	11	>4	<1000	17	>16	0	>4/76	17	>128
FSL H7-0353	13	>16	0	>64	1	21	<0.5	8	28	<0.25	1	28	4	0.25	1	8	8	1	15	<0.5	<1000	17	<2	0	>4/76	17	<1
FSL M7-1345	15	8	8	64	0.25	21	<0.5	8	20	<0.25	1	29	<2	0.25	0.5	4	8	2	19	<0.5	<1000	18	<2	0	>4/76	17	<1
FSL M7-0322	16	>16	10	64	0.5	23	<0.5	4	23	<0.25	1	29	<2	0.25	1	8	8	2	19	<0.5	<1000	18	<2	0	>4/76	17	<1
FSL K6-0220	15	8	0	32	0.12	23	<0.5	8	26	<0.25	1	29	<2	0.25	0.5	2	8	0.5	24	<0.5	<1000	20	<2	0	>4/76	17	<1
FSL M7-0690	27	>16	14	64	0.5	28	<0.5	8	27	<0.25	1	40	<2	0.25	1	8	8	1	20	<0.5	<1000	21	<2	0	>4/76	17	<1
FSL P2-0558	13	>16	0	>64	1	28	1	8	30	<0.25	1	29	4	1	2	8	8	2	20	<0.5	<1000	24	<2	0	>4/76	17	2
FSL W7-1328	22	>16	24	16	0.5	33	<0.5	8	20	0.5	1	33	<2	0.25	1	4	8	1	28	<0.5	<1000	0	>16	12	>4/76	17	<1
FSL H7-0683	14	>16	10	16	2	23	<0.5	2	22	<0.25	1	34	16	0.25	1	8	8	1	16	<0.5	<1000	17	<2	12	>4/76	17	<1
FSL K6-0267	16	2	11	64	2	26	<0.5	8	26	<0.25	1	30	<2	0.25	2	2	8	1	20	<0.5	<1000	21	<2	17	>4/76	17	<1
FSL W7-1101	12	>16	7	>64	2	26	<0.5	8	19	2	1	29	<2	0.25	1	8	8	2	14	<0.5	<1000	26	4	19	>4/76	17	2
FSL H7-0344	17	>16	0	32	0.5	24	<0.5	8	26	<0.25	1	34	<2	0.25	1	8	8	1	16	<0.5	<1000	14	<2	0	>4/76	18	2
FSL H8-0485	24	>16	10	>64	0.25	18	<0.5	8	29	<0.25	1	26	<2	0.25	0.5	8	8	2	15	<0.5	<1000	16	<2	0	>4/76	18	<1

FSL H7-0926	15	>16	10	>64	0.12	21	<0.5	8	24	<0.25	1	32	<2	0.25	0.5	8	8	1	12	<0.5	<1000	17	<2	0	>4/76	18	<1
FSL K6-0040	15	>16	0	>64	1	21	<0.5	4	30	<0.25	1	20	<2	0.25	1	8	8	1	14	<0.5	<1000	17	<2	0	>4/76	18	<1
FSL M7-1472	16	>16	11	32	0.5	20	<0.5	8	26	<0.25	1	34	<2	0.25	1	8	8	2	17	<0.5	<1000	17	<2	0	>4/76	18	2
FSL P4-0569	18	>16	0	>64	0.5	26	<0.5	8	33	<0.25	1	31	<2	0.25	1	8	8	1	20	<0.5	<1000	18	<2	0	>4/76	18	<1
FSL R5-0920	20	>16	12	>64	0.25	22	<0.5	8	25	<0.25	1	27	4	0.25	1	8	8	1	21	<0.5	<1000	18	<2	0	>4/76	18	<1
FSL R7-0282	0	>16	0	>64	1	25	<0.5	8	30	<0.25	1	32	<2	0.25	1	8	8	1	23	<0.5	<1000	20	<2	0	>4/76	18	2
FSL P2-0235	15	>16	8	>64	1	25	<0.5	8	32	<0.25	1	31	4	0.5	1	8	8	2	21	<0.5	<1000	21	<2	0	>4/76	18	2
FSL M7-1219	15	>16	9	>64	0.5	21	<0.5	8	30	<0.25	1	33	<2	0.25	0.5	8	8	0.5	17	<0.5	<1000	19	<2	18	>4/76	18	2
FSL M8-0473	20	>16	14	64	0.5	30	<0.5	8	33	<0.25	1	32	4	0.25	1	8	8	1	23	<0.5	<1000	18	<2	0	>4/76	19	2
FSL W8-0824	20	>16	12	>64	1	28	<0.5	8	34	<0.25	1	32	4	0.5	1	8	8	2	20	2	<1000	20	<2	0	>4/76	19	2
FSL R5-0859	17	>16	0	>64	1	26	<0.5	8	27	0.5	1	31	16	1	1	8	8	1	23	1	<1000	22	<2	0	>4/76	19	2
FSL M7-0938	20	>16	8	>64	0.5	25	<0.5	8	31	<0.25	1	30	4	0.25	2	8	8	1	18	<0.5	<1000	23	<2	0	>4/76	19	2
FSL W8-0520	18	>16	20	32	0.5	33	2	8	37	2	1	31	16	2	2	8	8	2	24	>4	<1000	30	<2	0	>4/76	19	2
FSL M7-0109	18	>16	0	64	0.25	20	<0.5	8	29	<0.25	1	28	8	0.25	1	8	8	2	18	<0.5	<1000	20	<2	14	>4/76	19	<1
FSL H8-0049	14	>16	8	>64	0.5	25	<0.5	4	25	<0.25	1	30	4	1	1	8	8	1	17	<0.5	<1000	19	<2	0	>4/76	20	<1
FSL R7-0047	18	0.5	7	32	0.25	23	<0.5	8	28	1	1	26	8	2	1	4	1	2	18	2	<1000	19	<2	0	>4/76	20	<1
FSL P2-0021	13	>16	7	64	0.25	22	<0.5	8	31	<0.25	1	28	<2	0.25	0.5	0.25	8	0.5	22	<0.5	<1000	20	<2	0	>4/76	20	<1
FSL R5-0708	18	>16	12	64	0.12	27	<0.5	8	25	1	1	34	8	1	0.5	8	8	1	18	4	<1000	21	<2	0	>4/76	20	<1
FSL W8-0003	27	>16	20	<8	1	27	<0.5	8	28	2	1	32	16	1	2	8	8	2	23	1	<1000	32	<2	0	>4/76	20	2

FSL K6-0069	23	>16	12	32	0.5	31	<0.5	4	36	<0.25	1	37	4	0.25	1	8	8	1	23	<0.5	<1000	23	<2	10	>4/76	20	2
FSL W7-1108	14	>16	8	>64	0.12	21	<0.5	8	20	<0.25	1	21	<2	0.25	1	8	8	2	15	<0.5	<1000	25	<2	15	>4/76	20	<1
FSL W8-0275	3	>16	15	32	1	28	<0.5	8	29	2	1	36	<2	0.25	1	8	8	4	22	<0.5	<1000	10	>16	20	>4/76	20	2
FSL P4-0260	20	>16	14	64	0.25	27	<0.5	8	34	<0.25	1	38	<2	0.25	0.5	2	8	1	21	<0.5	<1000	26	<2	23	>4/76	20	2
FSL J3-0123	15	>16	8	>64	0.5	29	<0.5	8	25	<0.25	1	31	4	0.25	0.5	8	8	1	17	<0.5	<1000	25	<2	16	>4/76	21	<1
FSL H8-0545	26	8	17	16	0.25	34	<0.5	2	35	<0.25	1	38	<2	0.25	0.5	1	8	0.5	17	<0.5	<1000	34	<2	17	>4/76	21	<1
FSL W7-1334	25	>16	11	>64	0.5	31	1	8	29	1	1	40	4	0.5	1	8	8	1	20	4	<1000	24	<2	20	>4/76	21	2
FSL E2-0214	12	>16	20	>64	2	44	<0.5	4	33	<0.25	1	43	<2	0.25	1	8	8	2	27	1	<1000	25	<2	24	>4/76	21	<1
FSL M8-0214	26	>16	16	>64	0.5	34	<0.5	8	27	4	1	42	<2	0.25	1	8	8	2	24	<0.5	<1000	31	<2	0	>4/76	22	2
FSL H8-0534	28	8	10	>64	2	34	<0.5	8	38	<0.25	1	35	4	0.25	1	8	8	2	33	<0.5	<1000	28	<2	16	>4/76	22	<1
FSL K6-0268	26	>16	15	>64	0.25	34	<0.5	4	27	<0.25	1	44	<2	0.25	1	8	8	1	26	<0.5	<1000	28	<2	28	>4/76	23	<1
FSL K6-1030	45	>16	20	64	1	32	<0.5	4	36	1	1	40	<2	0.25	0.5	8	8	2	31	<0.5	<1000	25	<2	0	>4/76	25	2

[a] Inhibition zone interpreted using CLSI M100 *Staphylococcus* spp. breakpoints.
[b] Resistance undetermined due to the lack of defined clinical breakpoints.
[c] Minimum inhibitory concentration interpreted with CLSI M45 CLSI *Bacillus* spp. breakpoints.
[d] Minimum inhibitory concentrations interpreted with EUCAST v12.00 *Bacillus* spp. breakpoints.

SUPPLEMENTAL TABLE S3: Antimicrobial resistance genes (ARGs) detected in draft genomes of 85 *B. cereus* group isolates using ABRicate with MEGARes 2.0 database or ResFinder database, and ARIBA with MEGARes 2.0 or ResFinder database.

Isolate	ABRICATE – MEGARES 2.0																ARIBA – MEGARES 2.0[a]											ABRICATE - RESFINDER							ARIBA – RESFINDER[a]			
	BC	BCII	BLA1	BLAZ	FOSB	MPH	MPHB	RPH	SAT	blaTEM	TET45	TETL	VANRA	VANSA	VANYA	VANYF	BCII	BCII+	BLA1+	BLAZ_2	FOSB_3	FOSB_4	MPH	MPH-	SAT_1	TETL_1	VANRA	blaTEM-116_1	blaZ_12	fosB1_1	fosB2_1	fosB_5	tet(45)_1	tet(L)_2	blaZ.match	fosB1.match	tet_L__1.match	tet(L)_match
FSL K6-0040	100	100	.	98.74	100	.	100	.	98.74	.	.	.	.	.	.	94.2	yes	no	yes	yes	no	yes	no	yes	yes	no	no	.	98.74	100	.	.	.	.	yes	yes	no	no
FSL W8-0640	99.46	100	.	98.74	100	.	.	.	.	.	.	.	100	99.74	90.56	92.75;94.20	yes	no	yes	no	no	yes	no	no	no	no	no	.	98.74	100	.	.	.	.	no	yes	no	no
FSL H8-0488	99.46	100	.	.	99.76	100	.	.	98.74	.	.	.	.	.	.	92.59	no	no	yes	no	no	no	yes	no	yes	no	no	.	.	99.76	.	.	.	.	no	no	no	no
FSL R5-0184	100	100	.	99.47	100	.	.	.	98.38	.	.	.	100	99.74	.	94.20;94.20	yes	no	yes	no	no	yes	no	no	yes	no	yes	.	99.47	100	.	.	.	.	no	yes	no	no
FSL W8-0169	99.46	100	.	99.68	99.76	.	.	.	.	.	.	.	100	99.74	.	94.04;94.20	no	yes	no	no	no	no	no	no	no	no	no	.	99.68	99.76	.	.	.	.	no	no	no	no
FSL K6-0067	.	100	99.46	.	99.76	.	.	.	98.38	.	.	.	.	.	.	94.2	no	yes	no	no	no	no	no	no	no	no	no	.	.	99.76	.	.	.	.	no	no	no	no
FSL K6-0069	.	99.74	99.78	.	99.76	.	.	.	.	.	.	.	.	.	.	92.75	no	yes	no	no	no	no	no	no	no	no	no	.	.	99.76	.	.	.	.	no	no	no	no
FSL K6-0073	100	100	.	.	100	.	.	.	.	.	.	.	100	99.74	.	94.20;94.20	yes	no	yes	no	no	yes	no	no	no	no	no	.	.	100	.	.	.	.	no	yes	no	no
FSL H7-0353	99.46	100	.	99.68	99.76	.	.	.	.	.	.	.	.	.	.	94.2	no	yes	yes	no	no	no	no	no	no	no	no	.	99.68	99.76	.	.	.	.	no	no	no	no
FSL M8-0117	.	100	100	.	99.76	.	.	.	98.38	.	.	.	.	.	.	94.2	no	yes	yes	no	no	no	no	no	no	no	no	.	.	99.76	.	.	.	.	no	no	no	no
FSL W8-0003	.	100	99.46	.	99.76;100.00	100	.	.	.	.	.	.	.	.	.	.	no	yes	yes	no	yes	no	no	no	no	no	no	.	.	99.76	.	100	.	.	no	no	no	no
FSL W8-0050	99.46	99.61	.	.	100.00;99.76	.	.	.	100	.	.	.	.	.	.	94.2	no	no	yes	no	yes	no	no	no	yes	no	no	.	.	99.76	.	100	.	.	no	no	no	no
FSL H8-0032	99.46	100	.	99.68	99.76	.	.	.	.	.	.	.	.	.	.	94.2	no	yes	yes	no	no	no	no	no	no	no	no	.	99.68	99.76	.	.	.	.	no	no	no	no
FSL W8-0268	100	100	.	.	100	.	.	.	98.38	.	.	.	.	.	.	94.2	yes	no	yes	no	no	yes	no	no	yes	no	no	.	.	100	.	.	.	.	no	yes	no	no
FSL W8-0275	.	100	99.46	.	99.76	100	.	.	.	.	99.64	.	.	.	.	94.2	no	yes	yes	no	no	no	no	no	no	no	no	.	.	99.76	.	.	99.64	.	no	no	no	no
FSL W8-0483	.	100	99.46	.	99.76	100	.	.	.	.	.	.	.	.	.	94.2	no	yes	yes	no	no	no	no	no	no	no	no	.	.	99.76	.	.	.	.	no	no	no	no
FSL W8-0520	.	99.61	99.46	.	99.76	.	.	.	98.38	.	.	.	.	.	.	94.2	no	no	yes	no	no	no	no	no	no	no	no	.	.	99.76	.	.	.	.	no	no	no	no
FSL W8-0523	.	99.61	99.46	.	99.76	.	.	.	98.38	.	.	.	.	.	.	94.2	no	no	yes	no	no	no	no	no	no	no	no	.	.	99.76	.	.	.	.	no	no	no	no
FSL M7-0044	99.46	100	.	99.68	99.76	.	.	.	.	.	.	.	.	.	.	94.2	no	yes	yes	no	no	no	no	no	no	no	no	.	99.68	99.76	.	.	.	.	no	no	no	no
FSL W8-0824	100	100	.	.	100	.	.	.	100	.	.	.	.	.	.	94.2	yes	no	yes	no	no	yes	no	no	yes	no	no	.	.	100	.	.	.	.	no	yes	no	no

FSL W8-0932	.	99.61	100	.	99.76	100	.	.	.	98.38	.	.	.	.	.	94.2	no	yes	yes	no	no	no	no	no	no	no	no	90.24	.	99.76	.	.	.	.	no	no	no	no
FSL M7-0938	99.46	100	.	99.68	99.76	.	.	.	.	.	.	.	.	.	.	94.2	no	yes	yes	no	no	no	no	no	no	no	no	.	99.68	99.76	.	.	.	.	no	no	no	no
FSL M7-1251	99.46	100	.	99.68	99.76	.	.	.	98.38	.	.	.	.	.	.	93.24	no	yes	yes	no	no	no	no	no	yes	no	no	.	99.68	99.76	.	.	.	.	no	no	no	no
FSL P4-0569	99.46	100	.	99.68	99.76	.	.	.	.	.	.	.	.	.	.	94.2	no	yes	yes	no	no	no	no	no	no	no	no	.	99.68	99.76	.	.	.	.	no	no	no	no
FSL M7-0690	99.46	100	.	99.68	99.76	.	.	.	.	.	.	.	.	.	.	94.2	no	yes	yes	no	no	no	no	no	no	no	no	.	99.68	99.76	.	.	.	.	no	no	no	no
FSL H8-0049	99.46	100	.	99.68	99.76	.	.	.	.	.	.	.	.	.	.	94.2	no	yes	yes	no	no	no	no	no	no	no	no	.	99.68	99.76	.	.	.	.	no	no	no	no
FSL H7-0444	99.46	100	.	99.68	99.76	.	.	.	.	.	.	.	.	.	.	94.2	no	yes	yes	no	no	no	no	no	no	no	no	.	99.68	99.76	.	.	.	.	no	no	no	no
FSL H7-0926	99.46	100	.	99.79	99.76	.	.	.	.	.	.	.	.	.	.	92.75	no	yes	no	no	no	no	no	no	no	no	no	.	99.79	99.76	.	.	.	.	no	no	no	no
FSL P2-0415	99.46	100	.	.	99.76	.	.	.	.	.	.	.	100	99.74	.	94.20;94.20	no	yes	yes	no	no	no	no	no	no	no	no	.	.	99.76	.	.	.	.	no	no	no	no
FSL P2-0558	99.46	100	.	.	99.76	.	.	.	.	.	.	.	100	99.74	.	94.20;94.20	no	yes	yes	no	no	no	no	no	no	no	no	.	.	99.76	.	.	.	.	no	no	no	no
FSL H8-0482	99.46	100	.	99.79	99.76	100	.	.	98.74	.	.	.	.	.	.	92.59	no	no	yes	no	no	no	yes	no	yes	no	no	.	99.79	99.76	.	.	.	.	no	no	no	no
FSL K6-0220	.	.	.	.	95.92	.	.	97.47	.	.	.	.	.	.	.	.	no	no	no	no	no	no	no	no	no	no	no	.	.	95.92	.	.	.	.	no	no	no	no
FSL K6-0267	99.46	100	.	99.79	99.76	.	.	.	.	.	.	.	.	.	.	.	no	yes	no	no	no	no	no	no	no	no	no	.	99.79	99.76	.	.	.	.	no	no	no	no
FSL H7-0611	.	100	100	.	99.76	.	.	.	.	.	.	.	.	.	.	92.75	no	yes	no	no	no	no	no	no	no	no	no	.	.	99.76	.	.	.	.	no	no	no	no
FSL H8-0545	.	100	100	99.79	99.76	.	.	.	.	.	.	.	.	.	.	.	no	yes	no	no	no	no	no	no	no	no	no	.	99.79	99.76	.	.	.	.	no	no	no	no
FSL H7-0683	99.46	100	.	99.79	99.52; 100.00	.	.	.	.	.	.	.	.	.	.	.	no	yes	yes	no	yes	no	no	no	no	no	no	.	99.79	99.52	.	100	.	.	no	no	no	no
FSL K6-1142	.	.	.	.	95.92	.	.	99.96	.	.	.	.	.	.	.	.	no	no	no	no	no	no	no	no	no	no	no	.	.	95.92	.	.	.	.	no	no	no	no
FSL K6-1030	100	100	.	.	100	.	.	.	98.38	.	.	.	.	.	.	94.2	yes	no	yes	no	no	yes	no	no	yes	no	no	.	.	100	.	.	.	.	no	yes	no	no
FSL H8-0481	.	.	.	.	95.92	.	.	99.96	.	.	.	.	.	.	.	.	no	no	no	no	no	no	no	no	no	no	no	.	.	95.92	.	.	.	.	no	no	no	no
FSL E2-0214	99.46	100	.	99.79	99.52	.	.	.	.	.	.	.	.	.	.	.	no	yes	yes	no	no	no	no	no	no	no	no	.	99.79	99.52	.	.	.	.	no	no	no	no
FSL H7-0909	99.46	100	.	99.79	99.52; 100.00	.	.	.	.	.	.	.	.	.	.	.	no	yes	yes	no	yes	no	no	no	no	no	no	.	99.79	99.52	.	100	.	.	no	no	no	no
FSL H7-0676	99.78	100	.	99.79	99.52	.	.	.	.	.	.	.	.	.	.	.	no	yes	yes	no	no	no	no	no	no	no	no	.	99.79	99.52	.	.	.	.	no	no	no	no
FSL H8-0063	.	100	100	.	99.52	.	.	.	98.2	.	.	.	.	.	.	.	no	no	no	no	no	no	no	no	no	no	no	.	.	99.52	.	.	.	.	no	no	no	no
FSL H8-0534	.	.	.	.	95.92	.	.	97.47	.	.	.	.	.	.	.	.	no	no	no	no	no	no	no	no	no	no	no	.	.	95.92	.	.	.	.	no	no	no	no

FSL F4-0079	99.46	100	.	99.79	99.76	.	.	.	.	.	.	.	.	.	.	92.75	no	yes	yes	no	no	no	no	no	no	no	no	.	99.79	99.76	.	.	.	.	no	no	no	no
FSL H8-0492	99.46	100	.	99.79	99.76	.	.	.	98.38	.	.	.	.	.	.	92.75	no	no	yes	no	no	no	no	no	yes	no	no	.	99.79	99.76	.	.	.	.	no	no	no	no
FSL M7-0053	99.46	100	.	99.79	99.76	.	.	.	.	.	.	.	.	.	.	.	no	yes	no	no	no	no	no	no	no	no	no	.	99.79	99.76	.	.	.	.	no	no	no	no
FSL K6-3163	.	100	.	99.79	100	.	.	.	98.38	.	.	.	.	.	.	94.2	yes	no	no	no	no	yes	no	no	yes	no	no	.	99.79	100	.	.	.	.	no	yes	no	no
FSL W7-1328	.	.	100	.	99.76	.	.	.	.	.	.	100	100	99.74	.	94.20;94.20	no	no	yes	no	no	no	no	no	no	yes	no	.	.	99.76	.	.	.	100	no	no	yes	yes
FSL R7-0047	.	100	.	99.79	99.52	.	.	.	.	.	.	.	.	.	.	.	no	yes	no	no	no	no	no	no	no	no	no	.	99.79	99.52	.	.	.	.	no	no	no	no
FSL R7-0282	100	100	.	.	100	.	.	.	98.38	.	.	.	.	.	.	94.2	yes	no	yes	no	no	yes	no	no	yes	no	no	.	.	100	.	.	.	.	no	yes	no	no
FSL R7-0117	100	100	.	.	100	.	.	.	98.38	.	.	.	.	.	.	94.2	yes	yes	yes	no	no	yes	no	no	no	no	no	.	.	100	.	.	.	.	no	yes	no	no
FSL R5-0920	99.46	100	.	99.79	99.76	.	.	.	98.38	.	.	.	.	.	.	92.91	no	yes	yes	no	no	no	no	no	yes	no	no	.	99.79	99.76	.	.	.	.	no	no	no	no
FSL P4-0488	99.46	100	.	99.79	99.52; 100.00	.	.	.	.	.	.	.	.	.	.	.	no	no	yes	no	yes	no	no	no	no	no	no	.	99.79	99.52	.	100	.	.	no	no	no	no
FSL R5-0859	100	100	.	.	100	.	.	.	98.38	.	.	.	.	.	.	94.2	yes	no	yes	no	no	yes	no	no	yes	no	no	.	.	100	.	.	.	.	no	no	no	no
FSL R5-0832	100	100	.	.	100	.	.	.	98.38	.	.	.	.	.	.	94.2	yes	no	yes	no	no	yes	no	no	yes	no	no	.	.	100	.	.	.	.	no	yes	no	no
FSL M8-0214	99.46	100	.	99.79	99.76	100	.	.	98.74	.	.	.	.	.	.	92.59	no	no	yes	no	no	no	yes	no	yes	no	no	.	99.79	99.76	.	.	.	.	no	no	no	no
FSL M7-1345	.	100	99.78	99.79	99.76	.	.	.	98.2	.	.	.	.	.	.	.	no	no	yes	no	no	yes	no	no	no	no	no	.	99.79	99.76	.	.	.	.	no	yes	no	no
FSL M7-1090	.	100	99.46	99.79	99.52	.	.	.	.	.	.	.	.	.	.	94.2	no	yes	yes	no	no	no	no	no	no	no	no	.	99.79	99.52	.	.	.	.	no	no	no	no
FSL M7-1006	99.46	100	.	99.79	100.00;99.52	.	.	.	.	.	.	.	.	.	.	.	no	yes	no	no	yes	no	no	no	no	no	no	.	99.79	99.52	.	100	.	.	no	no	no	no
FSL P4-0260	.	100	100	.	99.76	.	.	.	.	.	.	.	.	.	.	.	no	yes	yes	no	no	no	no	no	no	no	no	.	.	99.76	.	.	.	.	no	no	no	no
FSL P2-0235	100	100	.	.	100	.	.	.	98.38	.	.	.	.	.	.	94.2	yes	no	yes	no	no	yes	no	no	no	no	no	.	.	100	.	.	.	.	no	yes	no	no
FSL P2-0021	.	100	100	.	99.76	.	.	.	98.2	.	.	.	.	.	.	.	no	no	no	no	no	no	no	no	no	no	no	.	.	99.76	.	.	.	.	no	no	no	no
FSL M7-0322	99.46	100	.	99.79	99.76	.	.	.	98.2	.	.	.	.	.	.	.	no	yes	yes	no	no	no	no	no	no	no	no	.	99.79	99.76	.	.	.	.	no	no	no	no
FSL M8-0139	99.46	100	.	.	100	.	.	.	.	.	.	.	.	.	.	.	yes	no	yes	no	no	yes	no	no	no	no	no	.	.	100	.	.	.	.	no	yes	no	no
FSL W7-1108	.	100	99.46	99.79	99.52	.	100	.	.	.	.	.	.	.	.	.	no	yes	no	no	no	no	no	yes	no	no	no	.	99.79	99.52	.	.	.	.	no	no	no	no
FSL M7-0109	99.46	100	.	99.79	100.00;99.52	.	.	.	.	.	.	.	.	.	.	.	no	yes	yes	no	yes	no	no	no	no	no	no	.	99.79	99.52	.	100	.	.	no	no	no	no

FSL J3-0123	.	100	100	99.79	99.76	.	.	.	98.2	.	.	.	.	.	.	.	no	yes	yes	no	no	yes	no	no	no	no	no	.	99.79	99.76	.	.	.	.	no	yes	no	no
FSL H8-0485	99.46	100	.	99.79	99.76	.	.	.	98.38; 98.20	.	.	.	.	.	.	92.75	no	yes	yes	no	no	no	no	no	yes	no	no	.	99.79	99.76	.	.	.	.	no	no	no	no
FSL R5-0708	99.46	100	.	99.79	99.52	.	.	.	.	.	.	.	.	.	.	.	no	no	no	no	no	no	no	no	no	no	no	.	99.79	99.52	.	.	.	.	no	no	no	no
FSL W8-0767	99.46	100	.	100	100	.	.	.	98.38	.	.	.	100	99.74	.	94.20;94.20	no	no	yes	yes	no	yes	no	no	yes	no	no	.	100	100	.	.	.	.	yes	yes	no	no
FSL K6-0042	.	.	.	.	95.92	.	.	99.96	.	.	.	.	.	.	.	.	no	no	no	no	no	no	no	no	no	no	no	.	.	95.92	.	.	.	.	no	no	no	no
FSL R5-0594	.	100	100	100	99.76	.	.	.	.	.	.	.	100	99.74	.	94.20;94.20	no	yes	yes	yes	no	no	no	no	no	no	no	.	100	99.76	.	.	.	.	yes	no	no	no
FSL R5-0585	99.46	100	.	96.32;98.74	100	.	.	.	98.38	.	.	.	.	.	.	94.2	yes	no	yes	no	no	yes	no	no	yes	no	no	.	96.32;98.74	100	.	.	.	.	no	yes	no	no
FSL K6-0268	100	.	.	98.74;96.32	100	100	.	.	98.38	.	.	.	.	.	.	94.2	no	no	yes	no	no	yes	yes	no	yes	no	no	.	98.74;96.32	100	.	.	.	.	no	yes	no	no
FSL H7-0344	.	100	100	.	99.76	.	.	.	.	.	.	.	.	.	.	92.75	no	yes	no	no	no	no	no	no	no	no	no	.	.	99.76	.	.	.	.	no	no	no	no
FSL W7-1101	100	.	.	98.74;96.32;99.79	100	100	.	.	98.38	.	.	.	.	.	.	94.2	no	no	yes	no	no	yes	yes	no	no	no	no	.	98.74;96.32;99.79	100	.	.	.	.	no	yes	no	no
FSL J3-0113	.	99.22	99.78	99.05;99.79	99.76	100	.	.	98.38; 98.02	.	.	.	.	.	.	92.75	no	yes	yes	yes	no	no	no	no	no	no	no	.	99.05;99.79	99.76	.	.	.	.	yes	no	no	no
FSL K6-0043	99.46	100	.	99.79;100.00	100	.	.	.	98.38	.	.	.	.	.	.	94.2	no	no	yes	no	no	yes	no	no	no	no	no	.	99.79;100.00	100	.	.	.	.	no	yes	no	no
FSL W7-1334	100	.	.	99.79;98.74;96.32	100	100	.	.	98.38	.	.	.	.	.	.	94.2	no	no	yes	no	no	yes	yes	no	yes	no	no	.	99.79;98.74;96.32	100	.	.	.	.	no	yes	no	no
FSL M8-0473	100	100	.	.	100	.	.	.	100	.	.	.	.	.	.	94.2	yes	no	yes	no	no	yes	no	no	no	no	no	.	.	100	.	.	.	.	no	yes	no	no
FSL R5-0811	100	100	.	.	100	.	.	.	98.38	.	.	.	.	.	.	94.2	yes	no	yes	no	no	yes	no	no	yes	no	no	.	.	100	.	.	.	.	no	yes	no	no
FSL M7-1472	.	100	99.46	99.89;99.79	99.76	.	.	.	.	.	.	.	.	.	.	.	no	yes	no	no	no	no	no	no	no	no	no	.	99.89;99.79	99.76	.	.	.	.	no	no	no	no
FSL M8-0091	.	100	99.46	99.89;99.79	100.00;99.76	.	.	.	.	.	.	.	.	.	.	92.75	no	yes	no	no	no	no	no	no	no	no	no	.	99.89;99.79	99.76	100	.	.	.	no	no	no	no
FSL M7-1219	.	100	100	.	99.76	.	.	.	.	.	.	.	.	.	.	.	no	no	no	no	no	yes	no	no	no	no	no	.	.	99.76	.	.	.	.	no	no	no	no

[a] Genes indicated with "yes" were detected with ≥ 90% coverage and identity.

SUPPLEMENTAL TABLE S4: Point mutations detected in the *rpoB* gene sequence of 85 *B. cereus* group isolates.

Isolate	MIC[a] (µg/ml)	AST[b]	Number of Point Mutations	I309V	E339A	V359S	V372I	T553S	Q559H	E582D	E583V	S588D	K640R	M650I	H677Q	V620T/I	E696D	N733D	E734E	V1103I	S1146I	D1147N	T1149K
FSL R7-0047	2	I	14	1	1	1	1	0	0	0	1	1	0	1	1	0	1	1	1	1	1	1	0
FSL W8-0824	2	I	3	0	0	0	0	0	1	1	0	0	1	0	0	0	0	0	0	0	0	0	0
FSL H8-0488	>4	R	6	1	1	0	1	0	0	0	0	1	0	0	0	0	0	0	0	1	0	0	1
FSL M8-0117	>4	R	2	0	0	0	0	0	0	1	0	0	1	0	0	0	0	0	0	0	0	0	0
FSL R5-0708	4	R	14	1	1	1	1	0	0	0	1	1	0	1	1	0	1	1	1	1	1	1	0
FSL W8-0169	4	R	2	0	0	0	0	1	0	0	0	0	0	0	0	0	0	0	0	0	0	0	1
FSL W7-1334	4	R	3	0	0	0	0	0	1	1	0	0	1	0	0	0	0	0	0	0	0	0	0
FSL W8-0520	>4	R	3	0	0	0	0	0	1	0	0	1	0	0	0	1	0	0	0	0	0	0	0
FSL W8-0523	>4	R	3	0	0	0	0	0	1	0	0	1	0	0	0	1	0	0	0	0	0	0	0
FSL H7-0344	<0.5	S	1	0	0	0	0	0	0	0	0	0	0	0	0	0	0	0	0	0	0	0	1
FSL H7-0353	<0.5	S	2	0	0	0	0	1	0	0	0	0	0	0	0	0	0	0	0	0	0	0	1
FSL H7-0444	<0.5	S	2	0	0	0	0	1	0	0	0	0	0	0	0	0	0	0	0	0	0	0	1
FSL H7-0611	<0.5	S	1	0	0	0	0	0	0	0	0	0	0	0	0	0	0	0	0	0	0	0	1
FSL H7-0676	<0.5	S	14	1	1	1	1	0	0	0	1	1	0	1	1	0	1	1	1	1	1	1	0
FSL H7-0683	<0.5	S	14	1	1	1	1	0	0	0	1	1	0	1	1	0	1	1	1	1	1	1	0
FSL H7-0909	<0.5	S	14	1	1	1	1	0	0	0	1	1	0	1	1	0	1	1	1	1	1	1	0
FSL H7-0926	<0.5	S	14	1	1	1	1	0	0	0	1	1	0	1	1	0	1	1	1	1	1	1	0
FSL H8-0032	<0.5	S	2	0	0	0	0	1	0	0	0	0	0	0	0	0	0	0	0	0	0	0	1
FSL H8-0049	<0.5	S	2	0	0	0	0	1	0	0	0	0	0	0	0	0	0	0	0	0	0	0	1
FSL H8-0063	<0.5	S	14	1	1	1	1	0	0	0	1	1	0	1	1	0	1	1	1	1	1	1	0
FSL H8-0482	<0.5	S	6	1	1	0	1	0	0	0	0	1	0	0	0	0	0	0	0	1	0	0	1

FSL H8-0485	<0.5	S	14	1	1	1	1	0	0	0	1	1	0	1	1	0	1	1	1	1	1	1	0
FSL H8-0492	<0.5	S	14	1	1	1	1	0	0	0	1	1	0	1	1	0	1	1	1	1	1	1	0
FSL H8-0545	<0.5	S	14	1	1	1	1	0	0	0	1	1	0	1	1	0	1	1	1	1	1	1	0
FSL J3-0113	<0.5	S	2	0	0	0	0	1	0	0	0	0	0	0	0	0	0	0	0	0	0	0	1
FSL J3-0123	<0.5	S	14	1	1	1	1	0	0	0	1	1	0	1	1	0	1	1	1	1	1	1	0
FSL K6-0040	<0.5	S	3	0	0	0	0	0	1	1	0	0	1	0	0	0	0	0	0	0	0	0	0
FSL K6-0043	<0.5	S	3	0	0	0	0	0	1	1	0	0	1	0	0	0	0	0	0	0	0	0	0
FSL K6-0067	<0.5	S	3	0	0	0	0	0	0	1	0	0	1	0	0	1	0	0	0	0	0	0	0
FSL M7-0044	<0.5	S	2	0	0	0	0	1	0	0	0	0	0	0	0	0	0	0	0	0	0	0	1
FSL M7-0053	<0.5	S	14	1	1	1	1	0	0	0	1	1	0	1	1	0	1	1	1	1	1	1	0
FSL M7-0109	<0.5	S	14	1	1	1	1	0	0	0	1	1	0	1	1	0	1	1	1	1	1	1	0
FSL M7-0322	<0.5	S	14	1	1	1	1	0	0	0	1	1	0	1	1	0	1	1	1	1	1	1	0
FSL M7-0938	<0.5	S	2	0	0	0	0	1	0	0	0	0	0	0	0	0	0	0	0	0	0	0	1
FSL M7-1090	<0.5	S	14	1	1	1	1	0	0	0	1	1	0	1	1	0	1	1	1	1	1	1	0
FSL M7-1219	<0.5	S	14	1	1	1	1	0	0	0	1	1	0	1	1	0	1	1	1	1	1	1	0
FSL M7-1251	<0.5	S	2	0	0	0	0	1	0	0	0	0	0	0	0	0	0	0	0	0	0	0	1
FSL M7-1345	<0.5	S	14	1	1	1	1	0	0	0	1	1	0	1	1	0	1	1	1	1	1	1	0
FSL M7-1472	<0.5	S	2	0	0	0	0	1	0	0	0	0	0	0	0	0	0	0	0	0	0	0	1
FSL M8-0091	<0.5	S	2	0	0	0	0	1	0	0	0	0	0	0	0	0	0	0	0	0	0	0	1
FSL P2-0415	<0.5	S	2	0	0	0	0	1	0	0	0	0	0	0	0	0	0	0	0	0	0	0	1
FSL P4-0488	<0.5	S	14	1	1	1	1	0	0	0	1	1	0	1	1	0	1	1	1	1	1	1	0
FSL R5-0184	<0.5	S	3	0	0	0	0	0	1	1	0	0	1	0	0	0	0	0	0	0	0	0	0
FSL R5-0585	<0.5	S	3	0	0	0	0	0	1	1	0	0	1	0	0	0	0	0	0	0	0	0	0
FSL R5-0594	<0.5	S	2	0	0	0	0	0	0	1	0	0	1	0	0	0	0	0	0	0	0	0	0

FSL R5-0811	<0.5	S	3	0	0	0	0	0	1	1	0	0	1	0	0	0	0	0	0	0	0	0	0
FSL R5-0832	1	S	3	0	0	0	0	0	1	1	0	0	1	0	0	0	0	0	0	0	0	0	0
FSL R7-0117	1	S	3	0	0	0	0	0	1	1	0	0	1	0	0	0	0	0	0	0	0	0	0
FSL W7-1101	<0.5	S	3	0	0	0	0	0	1	1	0	0	1	0	0	0	0	0	0	0	0	0	0
FSL W7-1108	<0.5	S	14	1	1	1	1	0	0	0	1	1	0	1	1	0	1	1	1	1	1	1	0
FSL W8-0050	<0.5	S	3	0	0	0	0	0	1	0	0	1	0	0	0	1	0	0	0	0	0	0	0
FSL W8-0268	<0.5	S	3	0	0	0	0	0	1	1	0	0	1	0	0	0	0	0	0	0	0	0	0
FSL W8-0483	<0.5	S	3	0	0	0	0	0	1	0	0	1	0	0	0	1	0	0	0	0	0	0	0
FSL W8-0640	<0.5	S	3	0	0	0	0	0	1	1	0	0	1	0	0	0	0	0	0	0	0	0	0
FSL W8-0767	<0.5	S	3	0	0	0	0	0	1	1	0	0	1	0	0	0	0	0	0	0	0	0	0
FSL W8-0932	<0.5	S	3	0	0	0	0	0	1	0	0	1	0	0	0	1	0	0	0	0	0	0	0
FSL E2-0214	1	S	14	1	1	1	1	0	0	0	1	1	0	1	1	0	1	1	1	1	1	1	0
FSL F4-0079	<0.5	S	14	1	1	1	1	0	0	0	1	1	0	1	1	0	1	1	1	1	1	1	0
FSL H8-0481	<0.5	S	7	1	0	1	0	0	1	0	0	1	0	0	0	1	0	1	1	0	0	0	0
FSL H8-0534	<0.5	S	6	0	0	1	0	0	1	0	0	1	0	0	0	1	0	1	1	0	0	0	0
FSL K6-0042	<0.5	S	7	1	0	1	0	0	1	0	0	1	0	0	0	1	0	1	1	0	0	0	0
FSL K6-0069	<0.5	S	1	0	0	0	0	0	0	0	0	0	0	0	0	0	0	0	0	0	0	0	1
FSL K6-0073	<0.5	S	3	0	0	0	0	0	1	1	0	0	1	0	0	0	0	0	0	0	0	0	0
FSL K6-0220	<0.5	S	6	0	0	1	0	0	1	0	0	1	0	0	0	1	0	1	1	0	0	0	0
FSL K6-0267	<0.5	S	14	1	1	1	1	0	0	0	1	1	0	1	1	0	1	1	1	1	1	1	0
FSL K6-0268	<0.5	S	3	0	0	0	0	0	1	1	0	0	1	0	0	0	0	0	0	0	0	0	0
FSL K6-1030	<0.5	S	3	0	0	0	0	0	1	1	0	0	1	0	0	0	0	0	0	0	0	0	0
FSL K6-1142	<0.5	S	7	1	0	1	0	0	1	0	0	1	0	0	0	1	0	1	1	0	0	0	0
FSL K6-3163	<0.5	S	3	0	0	0	0	0	1	1	0	0	1	0	0	0	0	0	0	0	0	0	0

FSL M7-0690	<0.5	S	2	0	0	0	0	1	0	0	0	0	0	0	0	0	0	0	0	0	0	0	1
FSL M7-1006	<0.5	S	14	1	1	1	1	0	0	0	1	1	0	1	1	0	1	1	1	1	1	1	0
FSL M8-0139	<0.5	S	3	0	0	0	0	0	1	1	0	0	1	0	0	0	0	0	0	0	0	0	0
FSL M8-0214	<0.5	S	6	1	1	0	1	0	0	0	0	1	0	0	0	0	0	0	0	1	0	0	1
FSL M8-0473	<0.5	S	3	0	0	0	0	0	1	1	0	0	1	0	0	0	0	0	0	0	0	0	0
FSL P2-0021	<0.5	S	14	1	1	1	1	0	0	0	1	1	0	1	1	0	1	1	1	1	1	1	0
FSL P2-0235	<0.5	S	3	0	0	0	0	0	1	1	0	0	1	0	0	0	0	0	0	0	0	0	0
FSL P2-0558	<0.5	S	2	0	0	0	0	1	0	0	0	0	0	0	0	0	0	0	0	0	0	0	1
FSL P4-0260	<0.5	S	14	1	1	1	1	0	0	0	1	1	0	1	1	0	1	1	1	1	1	1	0
FSL P4-0569	<0.5	S	2	0	0	0	0	1	0	0	0	0	0	0	0	0	0	0	0	0	0	0	1
FSL R5-0859	1	S	3	0	0	0	0	0	1	1	0	0	1	0	0	0	0	0	0	0	0	0	0
FSL R5-0920	<0.5	S	14	1	1	1	1	0	0	0	1	1	0	1	1	0	1	1	1	1	1	1	0
FSL R7-0282	<0.5	S	3	0	0	0	0	0	1	1	0	0	1	0	0	0	0	0	0	0	0	0	0
FSL W7-1328	<0.5	S	3	0	0	0	0	0	1	0	0	1	0	0	0	1	0	0	0	0	0	0	0
FSL W8-0003	1	S	3	0	0	0	0	0	1	0	0	1	0	0	0	1	0	0	0	0	0	0	0
FSL W8-0275	<0.5	S	3	0	0	0	0	0	1	0	0	1	0	0	0	1	0	0	0	0	0	0	0
**Total R**				2	2	1	2	1	3	2	1	4	2	1	1	2	1	1	1	2	1	1	2
**Total S**				29	26	29	26	14	31	22	24	37	22	24	24	12	24	29	29	26	24	24	19
**Total I**				1	1	1	1	0	1	1	1	1	1	1	1	0	1	1	1	1	1	1	0
**Total**				32	29	31	29	15	35	25	26	42	25	26	26	14	26	31	31	29	26	26	21

[a] MIC, minimum inhibitory concentration determined using the broth microdilution method.

[b] AST, antimicrobial susceptibility testing result determined using the CLSI M45 *Bacillus* spp. breakpoints.

SUPPLEMENTAL FIGURE S1 Distributions of minimum inhibitory concentrations (MICs) produced with broth microdilution method: ampicillin (A), ciprofloxacin (B), clindamycin (C), erythromycin (D), gentamicin (E), levofloxacin (F), linezolid (G), penicillin (H), rifampicin (I), tetracycline (J), trimethoprim-sulfamethoxazole (K), and vancomycin (L). The shades of grey-violet color indicate CLSI M45 breakpoints for *Bacillus* spp. for all antibiotics except linezolid, for which the EUCASTv12.0 *Bacillus* spp. breakpoints were used.

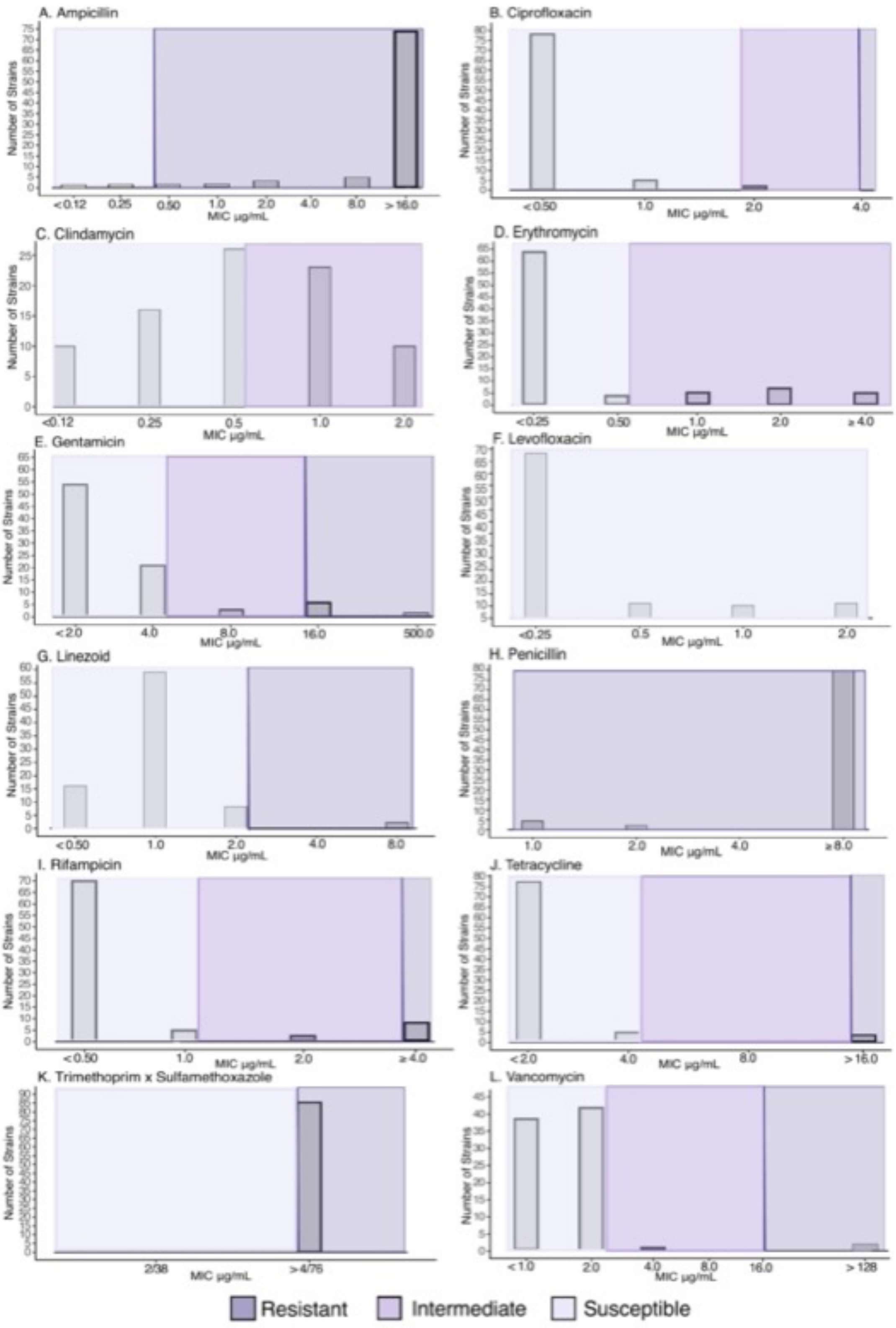
A. Ampicillin
B. Ciprofloxacin
C. Clindamycin
D. Erythromycin
E. Gentamicin
F. Levofloxacin
G. Linezoid
H. Penicillin
I. Rifampicin
J. Tetracycline
K. Trimethoprim x Sulfamethoxazole
L. Vancomycin
Number of Strains
MIC µg/mL
<0.12 0.25 0.50 1.0 2.0 4.0 8.0 >16.0
<0.50 1.0 2.0 4.0
<0.12 0.25 0.5 1.0 2.0
<0.25 0.50 1.0 2.0 ≥4.0
<2.0 4.0 8.0 16.0 500.0
<0.25 0.5 1.0 2.0
<0.50 1.0 2.0 4.0 8.0
1.0 2.0 4.0 ≥8.0
<0.50 1.0 2.0 ≥4.0
<2.0 4.0 8.0 >16.0
2/38 >4/76
<1.0 2.0 4.0 8.0 16.0 >128
Resistant
Intermediate
Susceptible

SUPPLEMENTAL FIGURE S2 Distributions of minimum inhibitory concentrations (MICs) for antibiotics for which resistance breakpoints were not available.

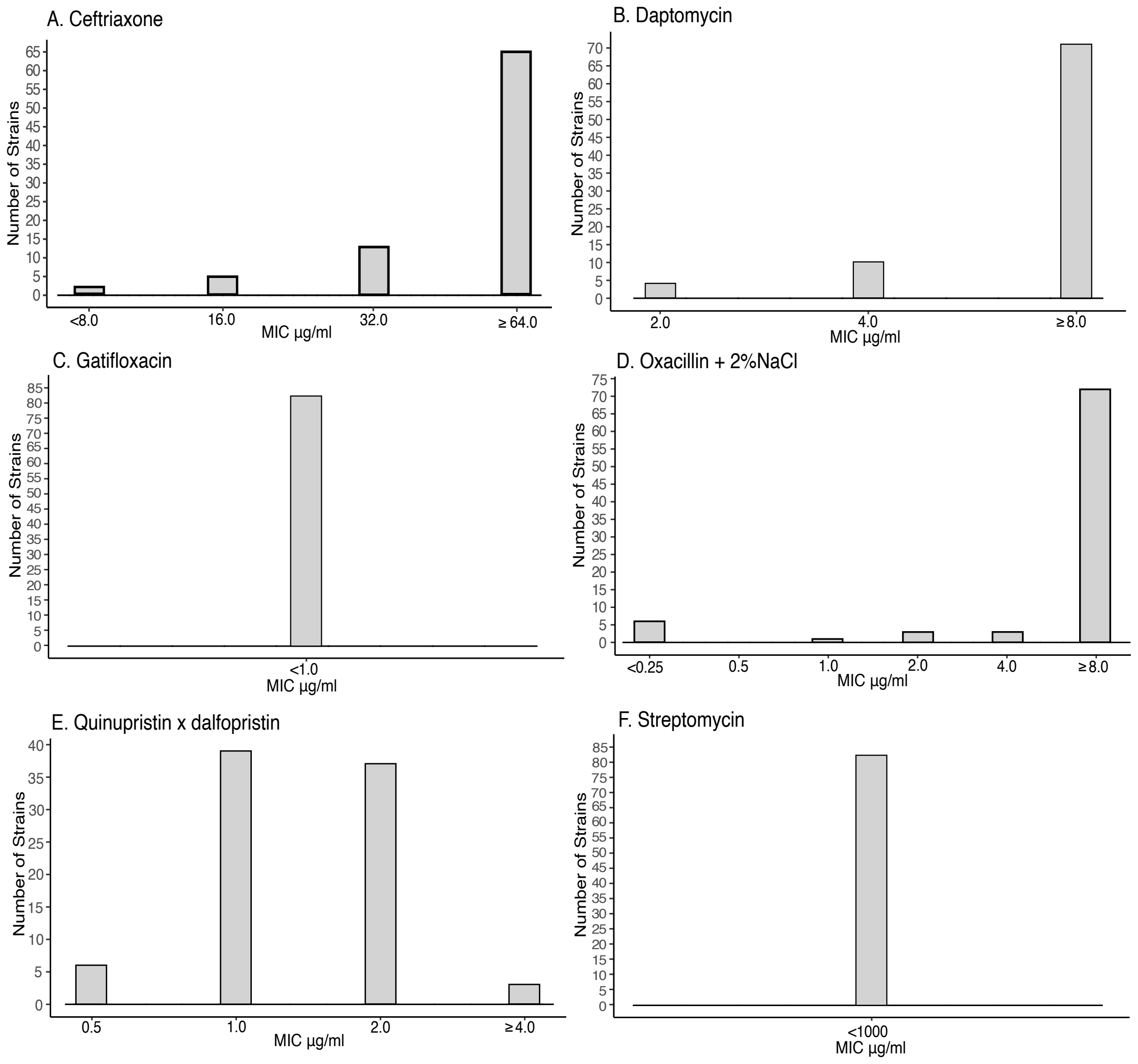

SUPPLEMENTAL FIGURE S3 Distribution of zones of inhibition determined using disk diffusion method for: ampicillin (A), ceftriaxone (B), ciprofloxacin (C), erythromycin (D), gentamicin (E), rifampicin (F), tetracycline (G), trimethoprim-sulfamethoxazole (H), and vancomycin (V). The shades of grey-turquoise color indicate CLSI M100 disk diffusion resistance breakpoints for *Staphylococcus* spp.

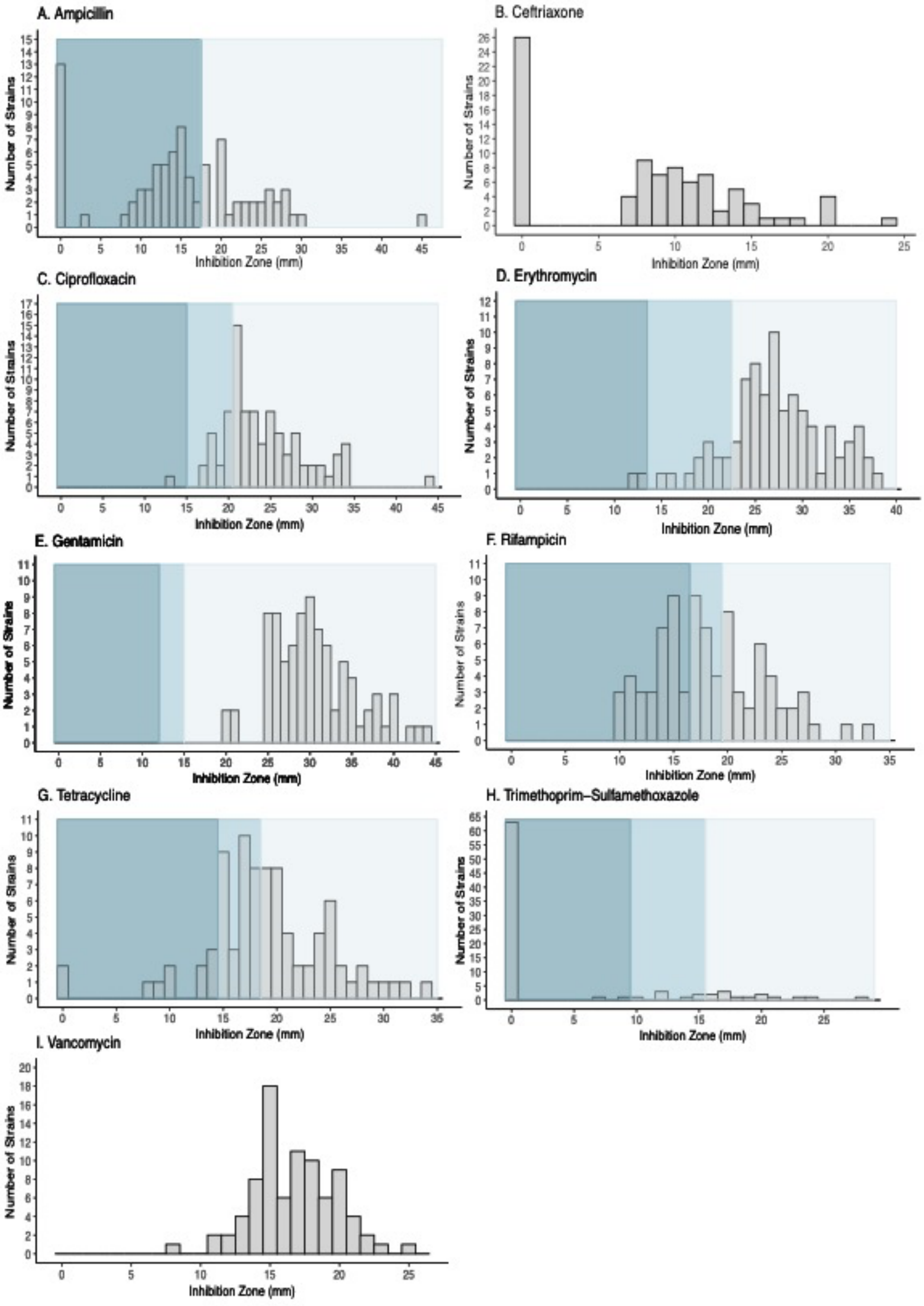
A. Ampicillin
Number of Strains
Inhibition Zone (mm)
B. Ceftriaxone
Number of Strains
Inhibition Zone (mm)
C. Ciprofloxacin
Number of Strains
Inhibition Zone (mm)
D. Erythromycin
Number of Strains
Inhibition Zone (mm)
E. Gentamicin
Number of Strains
Inhibition Zone (mm)
F. Rifampicin
Number of Strains
Inhibition Zone (mm)
G. Tetracycline
Number of Strains
Inhibition Zone (mm)
H. Trimethoprim–Sulfamethoxazole
Number of Strains
Inhibition Zone (mm)
I. Vancomycin
Number of Strains
Inhibition Zone (mm)
